# FLOT and CROSS chemotherapy regimens alter the frequency of CD27^+^ and CD69^+^ T cells in oesophagogastric adenocarcinomas: implications for combination with immunotherapy

**DOI:** 10.1007/s00432-022-04283-9

**Published:** 2022-08-20

**Authors:** Maria Davern, Noel E. Donlon, Andrew S. Sheppard, Klaudia D. Majcher, Fiona O’ Connell, Aisling B. Heeran, Malika Grant, Robert A. Farrell, Conall Hayes, Dara Bracken-Clarke, Melissa J. Conroy, Emma Foley, Dermot O’ Toole, Anshul Bhardwaj, Narayanasamy Ravi, John V. Reynolds, Stephen G. Maher, Jacintha O’ Sullivan, Joanne Lysaght

**Affiliations:** 1 Cancer Immunology and Immunotherapy Group, Department of Surgery, Trinity St. James’s Cancer Institute, Trinity Translational Medicine Institute, Trinity College, St. James’s Hospital Campus, Dublin 8, Ireland; 2 Department of Surgery, Trinity St. James’s Cancer Institute, Trinity Translational Medicine Institute, St. James’s Hospital, Trinity College Dublin, Dublin, Ireland; 3 Cancer Chemoradiation Group, Department of Surgery, Trinity St. James’s Cancer Institute, Trinity Translational Medicine Institute, St. James’s Hospital Campus, Dublin 8, Ireland

**Keywords:** Immune checkpoint inhibitors, DAMPs, Tumour microenvironment, CD69, CD27

## Abstract

Combining immunostimulatory chemotherapies with immunotherapy is an attractive strategy to enhance treatment responses in oesophagogastric junctional adenocarcinoma (OGJ). This study investigates the immunostimulatory properties of FLOT, CROSS and MAGIC chemotherapy regimens in the context of OGJ using in vitro and ex vivo models of the treatment-naïve and post-chemotherapy treated tumour microenvironment. FLOT and CROSS chemotherapy regimens increased surrogate markers of immunogenic cell death (HMGB1 and HLA-DR), whereas the MAGIC treatment regimen decreased HMGB1 and HLA-DR on OGJ cells (markedly for epirubicin). Tumour-infiltrating and circulating T cells had significantly lower CD27 expression and significantly higher CD69 expression post-FLOT and post-CROSS treatment. Similarly*,* the supernatant from FLOT- and CROSS-treated OGJ cell lines and from FLOT- and CROSS-treated OGJ biopsies cultured ex vivo also decreased CD27 and increased CD69 expression on T cells. Following 48 h treatment with post-FLOT and post-CROSS tumour conditioned media the frequency of CD69^+^ T cells in culture negatively correlated with the levels of soluble immunosuppressive pro-angiogenic factors in the conditioned media from ex vivo explants. Supernatant from FLOT- and CROSS-treated OGJ cell lines also increased the cytotoxic potential of healthy donor T cells ex vivo and enhanced OGJ patient-derived lymphocyte mediated-killing of OE33 cells ex vivo*.* Collectively, this data demonstrate that FLOT and CROSS chemotherapy regimens possess immunostimulatory properties, identifying these chemotherapy regimens as rational synergistic partners to test in combination with immunotherapy and determine if this combinatorial approach could boost anti-tumour immunity in OGJ patients and improve clinical outcomes.

**Supplementary Information:**

The online version contains supplementary material available at 10.1007/s00432-022-04283-9.

## Introduction

Oesophagogastric adenocarcinoma (OGJ) is the predominant subtype of oesophageal cancer in the Western world and its incidence continues to increase rapidly, largely attributed to increasing obesity rates (Pennathur et al. 2013). The standard of care for OGJ patients includes perioperative chemotherapy, neoadjuvant chemoradiotherapy and surgery (Pennathur et al. 2013). However, greater than two-thirds of OGJ patients do not achieve a complete pathological response following neo-adjuvant chemotherapy or neo-adjuvant chemoradiation (D’Journo and Thomas 2014; Smyth et al. 2017). While clinical outcomes in this patient cohort have improved, OGJ is still associated with a poor prognosis, with a 5-year overall survival rate of 15–25%, and approximately 45–50% for patients who are treated with curative intent (Morgan et al. 2020).

This underscores the clinical urgency to develop better treatment approaches to enhance the efficacy of neo-adjuvant chemotherapy regimens. Immunotherapy is now considered the fourth pillar of cancer therapy and has transformed treatment paradigms in many cancers. Immune checkpoint inhibitors (ICIs) reinvigorate anti-tumour immune responses, therefore, only patients with an intratumoural immune infiltrate and a pre-existing anti-tumour immune response will likely benefit from ICIs (Galon and Bruni 2019). Immunostimulatory chemotherapies are emerging as a valuable tool to convert ‘cold’ or immune-excluded tumours to ‘hot’ tumours by stimulating anti-tumour immune responses (Kershaw et al. 2013). Wherein, the addition of ICIs to these regimens prevents the exhaustion of chemotherapy-induced anti-tumour T cell responses resulting in synergistic effects (Kershaw et al. 2013).

Currently, clinical trials are ongoing in OGJ testing whether combining ICIs with chemotherapy regimens will improve the efficacy of chemotherapy (Davern and Lysaght 2020). Recent findings from the phase III CheckMate 649 trial demonstrated that combining nivolumab with first-line chemotherapy (FOLFOX and XELOX) in previously untreated OGJ patients (*n* = 1581) significantly improved overall survival in patients with a PD-L1 combined positive score of 5 or greater [14.4 months (nivolumab + chemotherapy arm) vs. 11.1 months (chemotherapy arm)] (Moehler et al. 2018). Furthermore, the nivolumab + chemotherapy arm also reduced the risk of death by 29% (HR, 0.71; 98.4% CI 0.59–0.86; *p* < 0.0001) (Moehler et al. 2018).

Indeed, conventional chemotherapy and radiotherapy regimens have profound effects on anti-tumour immunity. A recently published study by our group describes how radiotherapy promotes anti-tumour immunity increasing IL-21 and IL-23 cytokines and suppressing pro-tumourigenic pathways in the tumour microenvironment via downregulating the production of pro-angiogenic mediators (Donlon et al. 2022). However, the effects of chemotherapy on anti-tumour T cell immunity in OGJ remain unexplored, and this timely study aims to garner a greater understanding of first-line chemotherapy regimens which might alter T cell phenotypes in OGJ to either promote or enhance anti-tumour immunity.

Depending on the agent, chemotherapy can stimulate or suppress anti-tumour immunity (Wu and Waxman 2018). Therefore, it is critical to determine the immunostimulatory and immunosuppressive properties of each chemotherapy agent and as part of a regimen in the context of OGJ. The OGJ standard-of-care treatment consists of one of two main combination chemotherapy regimens given perioperatively (FLOT and MAGIC) or one chemoradiotherapy regimen (CROSS), given neo-adjuvantly (Al-Batran et al. 2008). The FLOT chemotherapy regimen includes the anti-metabolite 5-fluorouracil (5-FU), a platinum-based DNA intercalator oxaliplatin and a taxane anti-microtubule docetaxel (Al-Batran et al. 2008). The MAGIC chemotherapy regimen includes a topoisomerase inhibitor epirubicin, a platinum-based DNA intercalator cisplatin and an anti-metabolite 5-FU (ECF) or capecitabine (a pro-drug of 5-FU) (ECX) (Cunningham et al. 2006). The CROSS regimen comprises an anti-microtubule taxane paclitaxel and a platinum-based chemotherapy carboplatin in combination with daily 1.8 Gy doses of irradiation with a cumulative dose of 41.4 Gy (van Heijl et al. 2008). Nivolumab is also FDA approved in the adjuvant setting for those with completely resected OGJ tumours with residual pathologic disease who have received neoadjuvant chemoradiotherapy.

This timely study investigates the immunostimulatory and immunosuppressive properties of single agent chemotherapies and combination chemotherapy regimens used in the treatment of OGJ patients on T cell activation status and cytotoxicity. Combining immunostimulatory chemotherapies which are capable of inducing immunogenic cell death (ICD) with ICIs to synergistically enhance anti-tumour immunity is emerging as an attractive therapeutic tool (Galon and Bruni 2019; Pfirschke et al. 2016b). However, there is a wide range of chemotherapies that comprise the standard of care for OGJ patients and little is known regarding their ability to induce ICD, which is necessary to break peripheral tolerance. The potential of FLOT, CROSS or MAGIC chemotherapies to elicit ICD in OGJ cells or decrease surrogate markers of immunogenicity remains largely unknown. Furthermore, the OGJ tumour microenvironment (TME) consists of immunosuppressive cellular factors and soluble mediators which will be profoundly altered by chemoradiotherapy (CRT) treatment (Power et al. 2020; Lin et al. 2016), particularly within the treatment-resistant tumours which remain post-FLOT chemotherapy and post-CROSS CRT. Therefore, we also assessed T cell activation status in the treatment-naïve setting and post-FLOT and post-CROSS CRT setting by profiling OGJ patient-derived circulating T cells and tumour-infiltrating T cells.

The effect of the tumour conditioned media collected from ex vivo tumour explants post-FLOT and post-CROSS CRT on T cell activation and lymphocyte-mediated killing of OE33 cells were also investigated. This data will help guide the rational combination of immunostimulatory combination chemotherapy regimens with ICIs for treating OGJ.

## Materials and methods

### Ethical approval

Ethical approval was granted from the St. James’s Hospital Ethics Committee. All samples were collected with prior informed written consent for sample and data acquisition from patients attending St. James’s Hospital or from healthy donors. This study was carried out in accordance with the World Medical Association’s Declaration of Helsinki guidelines on medical research involving human subjects. Patient samples were pseudonymised in line with GDPR and data protection policies to protect the privacy and rights of the patients.

### Specimen collection

All patients involved in this study were enrolled from 2018 to 2020. Whole blood and tumour tissue biopsies were obtained from OGJ patients undergoing endoscopy at St. James’s hospital at time of diagnosis prior to initiation of any chemotherapy or radiotherapy. Post-FLOT chemotherapy-treated and post-CROSS chemoradiotherapy-treated whole blood and tumour tissue biopsies were obtained at time of surgical tumour resection. The cohort consisted of 17 males and 7 females, with an average age of 64.2 years. The patient demographics are detailed in Table 1 for the treatment-naïve and post-treatment cohort.

**Table 1:** Patient Demographics for treatment naive and post-treatment cohort

Age (years)	64.2
Sex ratio (M:F)	17:7
Diagnosis (no. patients)	
OGJ	22
Gastric adenocarcinoma	2
Clinical tumour stage (no. patients) (at time of diagnosis)	
T0	0
T1	3
T2	5
T3	16
T4	0
Clinical nodal status^a^ (no. patients) (at time of diagnosis)	
Positive	13
Negative	9
Received neoadjuvant FLOT	32% (7 patients)
Received neoadjuvant CROSS CRT	28% (6 patients)
Did not receive neoadjuvant treatment	40% (9 patients)
Response rate to neoadjuvant treatment	
FLOT	3/7 patients (42.85%) with a good response (TRG 1–2) and 4/7 patients (57.14%) with a poor response (TRG 3–5)
CROSS CRT	4/6 patients (66.66%) with a good response (TRG 1–2) and 2/6 patients (33.33%) with a poor response (TRG 3–5)

### OGJ tumour tissue digestion

Biopsies were enzymatically digested to perform OGJ cell phenotyping, as previously published (Kavanagh et al. 2019). Briefly, tissue was minced using a scalpel and digested in collagenase solution (2 mg/ml of collagenase type IV (Sigma) in Hanks Balanced Saline Solution (GE healthcare) supplemented with 4% (v/v) foetal bovine serum) at 37 °C and 1500 rpm on an orbital shaker. Tissue was filtered and washed with FACs buffer (PBS containing 1% foetal bovine serum and 0.01% sodium azide). Cells were then stained for flow cytometry.

### Cell line culture

Human cell lines (OE33, SK-GT-4) were purchased from European Collection of Cell Cultures. OE33, SK-GT-4 were grown in complete RPMI 1640 medium with 2 mM L-glutamine (Gibco) and supplemented with 1% (v/v) penicillin–streptomycin (50 U/ml penicillin 100 μg/ml streptomycin) and 10% (v/v) foetal bovine serum (Gibco) and maintained in a humidified chamber at 37 °C 5% CO_2_. Cell lines were tested regularly to ensure mycoplasma negativity. The OE33 cell line was established from a poorly differentiated stage IIA adenocarcinoma of the lower oesophagus (Barrett’s metaplasia) of a 73-year old female patient. SK-GT-4 cell line originated from a well differentiated oesophageal adenocarcinoma arising in Barrett’s epithelium from an 89-year old Caucasian male.

### Whole blood staining

Fluorochrome-conjugated antibodies were added to 100 µl blood at pre-optimized concentrations and incubated for 15 min at room temperature in the dark. Red cells were lysed using red blood cell lysing solution (Biolegend, USA), according to manufacturer’s recommendations and cells were washed twice with FACs buffer. Cells were fixed for 15 min in 1% paraformaldehyde solution (Santa Cruz Biotechnology, USA) prior to flow cytometric analysis.

### Flow cytometry staining

Trypsinised OE33 cells, SK-GT-4 cells or healthy donor PBMCs were stained with zombie aqua viability (Biolegend, USA) dye. Antibodies used for OE33 and SK-GT-4 cell lines and healthy donor PBMCs included: calreticulin-AF488 (Bio-techne, USA), HMGB-1-PE, MIC-A/B-APC, (Biolegend, USA), CD45RA-PE/Cy7, CD45RO-BV510, CD3-APC, CD3-PerCP, CD4-BV510, CD4-APC (Biolegend, USA), CD69-PE, CD62L-FITC, CD8-BV421 (BD Biosciences, USA) and CD27-APEefluor780 (eBioscience, USA). OE33 cells, SK-GT-4 cells and PBMCs were resuspended in FACs buffer and acquired using BD FACs CANTO II (BD Biosciences) using Diva software vX and analysed using FlowJo v10 software (TreeStar Inc.).

Whole blood and tumour tissue biopsies from OGJ patients were stained with zombie aqua viability (Biolegend, USA) and the following cell surface antibodies: CD45RA-PE/Cy7, CD62L-AF700, CD8-BV786, CD3-PEefluor610, CD69-BV605 (Biolegend, USA), CD27-APCefluor780 and CD4-PerCpCy5.5 (eBiosciences, USA). Cells were fixed with 1% paraformaldehyde solution, washed with FACs buffer, resuspended in FACs buffer and acquired using BD LSR Fortessa flow cytometer (BD Biosciences) using Diva software and analysed using FlowJo v10 software (TreeStar Inc.).

For intracellular cytokine staining, PBMCs were treated with PMA (10 ng/ml) and ionomycin (1 µg/ml) for the last 4 h of the incubation. Anti-CD107a-PE (BD Biosciences, USA) was added during stimulation. For the last 3 h of the incubation, PBMCs were treated with brefeldin A (10 µg/ml, eBiosciences). Cells were harvested, washed in FACs buffer and intracellular cytokines were assessed using a Fixation/Permeabilisation kit (BD Biosciences), as per manufacturer’s recommendations. Cells were stained with cell surface antibodies [CD8-BV421, CD3-APC or CD3-PerCP, CD4-PerCP, CD4-APC or CD4-BV510 (Biolegend, USA)] washed, permeabilised, and then stained for intracellular cytokines: IFN-γ-BV510, IL-17A-FITC, Granzyme B-PE/Cy7, Perforin-FITC BV510 (Biolegend, USA) and TNF-α-APC (BD Biosciences, USA). Cells were resuspended in FACs buffer and acquired using BD FACs CANTO II (BD Biosciences). Gating strategies for assessing DAMP expression on the surface of OGJ cells and activation marker expression and cytokine production by T cells are located in Fig. S1, Fig. S2 and Fig. S3, respectively.

### Generation of conditioned media

OE33 or SK-GT-4 cells were seeded at a density of 1 × 10^6^ cells/flask in T25 flasks and the media was changed the following day. When the flasks reached 40–50% confluency the cells were treated with a combination of chemotherapies that comprise the FLOT regimen (5-FU, oxaliplatin and docetaxel), the CROSS chemotherapy (CT) regimen (paclitaxel and carboplatin) or MAGIC chemotherapy regimen (epirubicin, cisplatin and 5-FU) or a vehicle control for 48 h [0.0001% DMSO, 0.001% H_2_O and 0.0001% NaCl (0.8% v/v)]. These combination doses were pre-optimised using a CCK8 assay to achieve 50% cell death [as carried out in Davern et al. (2021)]. The conditioned media (conditioned media) was harvested and stored at – 80 °C until required for experimentation.

Eleven OGJ resected tumour biopsies were also included. 5 OGJ tumour biopsies were taken post-FLOT treatment and 6 resected tumour biopsies taken post-CROSS treatment. OGJ tumour explants of ∼2–3 mm^3^ were transferred into 1 ml of serum-free M199 media (Gibco), supplemented with gentamicin and cultured for 24 h at 37 °C, 5% CO_2_. The resulting tumour conditioned media (TCM) was harvested and stored at − 80 °C until required for experimentation (Table 2).

**Table 2:** Patient Demographics for the generation of TCM

Age (years)	65.5
Sex ratio (M:F)	7:4
Diagnosis (no. patients)	
OGJ	11
Clinical tumour stage (no. patients) (at time of diagnosis)	
T0	1
T1	1
T2	1
T3	7
T4	1
Clinical nodal status (no. patients) (at time of diagnosis)	
Positive	6
Negative	5
Received neoadjuvant FLOT	45.5% (5/11 patients)
Received neoadjuvant CROSS CRT	54.5% (6/11 patients)
Response rate to neoadjuvant treatment	
FLOT	5/5 patients (100%) with a poor response (TRG 3–5)
CROSS CRT	2/6 patients (33.33%) with a good response (TRG 1–2) and 4/6 patients (66.66%) with a poor response (TRG 3–5)

Preparation of conditioned media for OE33-OGJ PBMC co-culture cytolysis assay: OE33 cells were treated for 48 h with vehicle (0.0001% DMSO and 0.0001% H_2_O), FLOT or CROSS CT chemotherapy regimens (IC_50_ doses as previously described in Davern et al. (2021)), washed twice to remove the chemotherapy drugs and the OE33 conditioned media was collected 48 h later.

### Co-culture of PBMCs with tumour ex vivo conditioned media or in vitro conditioned media

Healthy donor PBMCs were prepared from whole blood collected in EDTA Vacutainer tubes (BD Biosciences) by density gradient centrifugation over LymphoprepTM (Stemcell Technologies). PBMCs were plated at a concentration of 1 × 10^6^ cells per ml in complete RPMI media (10% FBS, 1% penstrep) and activated using plate bound anti-CD3 (10 μg/ml, Biolegend, USA) and anti-CD28 (10 μg/ml, Ancell, USA). PBMCs were concomitantly activated in the absence or presence of 50% OGJ cell conditioned media from 48 h vehicle-treated OE33/SK-GT-4 cells or FLOT, CROSS CT or MAGIC chemotherapy-treated OE33/SK-GT-4 cells or 50% tumour conditioned media (conditioned media) for 48 h. PBMCs were then harvested and stained for flow cytometry.

### Cytolysis assay

Cytolysis assay was carried out as previously demonstrated by Ding et al. (2019). OGJ PBMCs were isolated and expanded using plate bound anti-CD3 (10 μg/ml, Biolegend, USA) and anti-CD28 (10 μg/ml, Ancell, USA) and recombinant human IL-2 (Immunotools, Germany) for 5 days from treatment naïve OGJ blood using density gradient centrifugation (*n* = 6, duplicate technical replicates). OE33 cells were seeded at a density of 5 × 10^3^ cells/100 μl of media in a flat 96 well plate and incubated overnight at 37 °C, 5% CO_2_. The media was replaced and PBMCs were co-cultured with OE33 cells in an effector:target ratio of 5:1 for 48 h in the absence or presence of post-vehicle, post-FLOT or post-CROSS CT OE33 conditioned media (overall 1 in 2 dilution). PBMCs were also cultured alone to use as a control to account for an increase in viability due to their presence in the well. OE33 cells were also cultured alone. Following a 48 h co-culture 5 μl of CCK8 (Sigma, USA) was added to each well and the optical density at 450 nm and 650 nm (reference wavelength) was measured using the Versa Max microplate reader (Molecular Devices, Sunnyvale, CA, USA) to determine a viable cell number. Formula: (viability OE33 cell-lymphocyte co-culture)-(viability PBMCs alone)/(viability untreated OE33 cells alone) × 100 = % live cells.

### Statistical analysis

Data were analysed using GraphPad Prism 5 software (GraphPad Prism, San Diego, CA, USA) and was expressed as mean ± SEM. Statistical differences were analysed using a paired parametric Student’s *t*-test and unpaired parametric *t*-test where appropriate. Unpaired non-parametric *t*-test was conducted for patient sample data. Statistical significance was defined as *p* < 0.05.

## Results

### FLOT and CROSS chemotherapy regimens upregulate immunogenic marker expression on OGJ cells in vitro

**Fig. 1:**
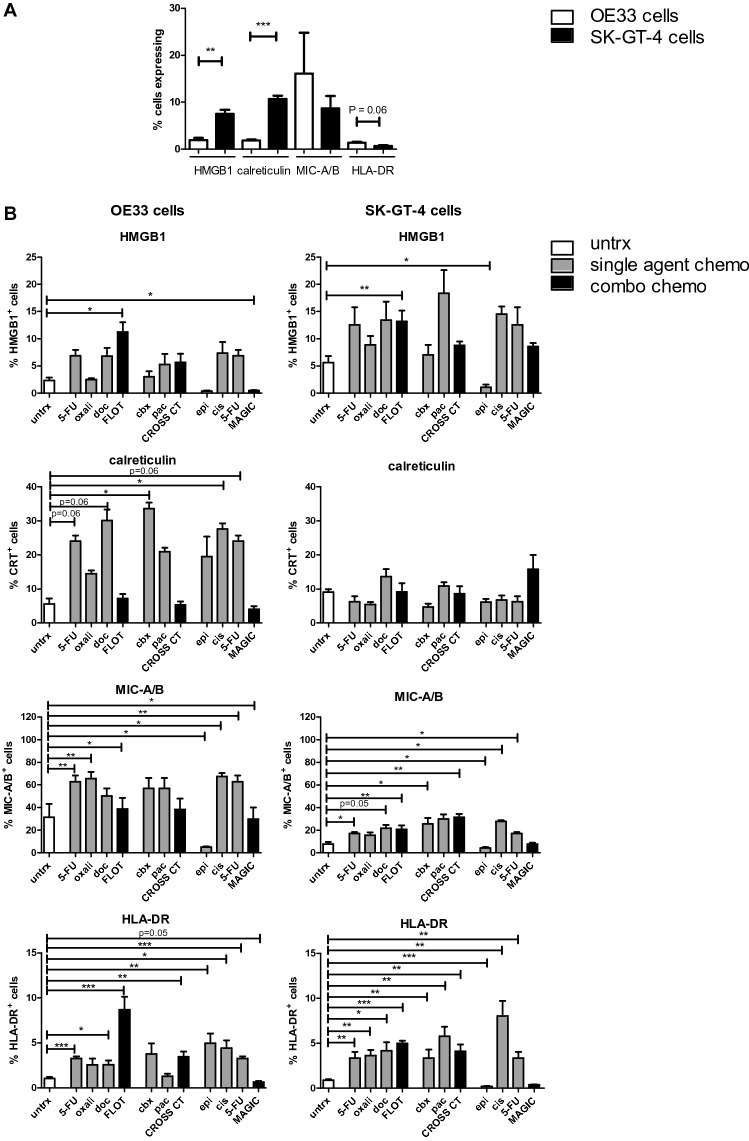
FLOT and CROSS chemotherapy regimens significantly upregulate DAMPs on the surface of viable OE33 and SK-GT-4 cells in vitro. **A** OE33 and SK-GT-4 cells were screened for the basal expression of HMGB1, calreticulin, MIC-A/B and HLA-DR by flow cytometry, *n* = 3, unpaired parametric *t*-test. **B** OE33 cells and SK-GT-4 cells were treated with clinically relevant single agent chemotherapies (single agent chemo) or combination chemotherapy regimens (combo chemo) FLOT, CROSS CT and MAGIC for 48 h. Surface expression of HMGB1, CRT, MIC-A/B and HLA-DR on viable OGJ cells was determined by flow cytometric analysis. Zombie viability dye was used to exclude dead cells. Paired parametric *t*-test compared to untreated (untrx) controls, *n* = 3, *p < 0.05, **p < 0.01, ***p < 0.001

As the immunogenicity of tumours vary significantly across patients, we investigated the basal immunogenicity of two OGJ cell lines (OE33 and SK-GT-4 cells). Both cell lines were screened by flow cytometry for the surface expression of damage-associated molecular patterns (DAMPs), which included high-mobility group box-1 protein (HMGB1), calreticulin, MIC-A/B, human leukocyte antigen complex–DR isotype (HLA-DR) and MHC class I-related chain-related gene A/B (MIC A/B) (Haabeth et al. 2018). SK-GT-4 cells had a significantly higher percentage expression of HMGB1 compared to OE33 cells (7.51 ± 0.9 vs. 1.95 ± 0.5%, *p* = 0.005) (Fig. 1A). Similarly, there was also a significantly higher percentage of SK-GT-4 cells expressing calreticulin basally compared to OE33 cells (10.69 ± 0.7 vs. 1.86 ± 0.2%, *p* = 0.0003) (Fig. 1A). There was no significant difference in the expression of MIC-A/B between OE33 cells and SK-GT-4 cells (Fig. 1A). There was no statistically significant difference between the percentage of OE33 cells and SK-GT-4 cells expressing HLA-DR (*p* = 0.06) (Fig. 1A).

An important therapeutic rationale for combining chemotherapy with ICIs is the chemotherapy-induction of ICD in tumour cells, thus generating an anti-tumour immune response whereby the addition of ICIs prevents exhaustion of the chemotherapy-induced anti-tumour immune response (Davern and Lysaght 2020). Therefore, we sought to investigate if the single agent chemotherapies used to treat OGJ and their combination as part of the FLOT, CROSS CT and MAGIC chemotherapy regimens upregulate markers of ICD in these two OGJ cells lines: the OE33 cell line which basally expresses lower levels of DAMPs than the SK-GT-4 cell line (Fig. 1A). OE33 and SK-GT-4 cells were treated with an IC_50_ dose of single agent chemotherapies or an IC_50_ dose of a combination chemotherapy regimen FLOT, CROSS CT or MAGIC for 48 h. The IC_50_ dose for each combination chemotherapy regimen comprised of lower doses of each single agent chemotherapy but resulted in an overall 50% reduction in viability [as previously described (Davern et al. 2021)].

One advantage to asking this question in vitro using the OE33 and SK-GT-4 tumour cell lines regarding if FLOT, CROSS CT or MAGIC could induce immunogenic tumour cell death in in tumour cells was that we could specifically determine if these regimens could induce immunogenic cell death without the added confounding variable of the complex tumour microenvironment which certainly would affect if these regimens could induce immunogenic cell death. However, to identify rational first-line chemotherapy regimens that could be ideal companion agents to combine with immunotherapy regimens we wanted to determine if these regimens had the capability to induce immunogenic tumour cell death in vitro. Further studies are indeed warranted to determine how the complex tumour microenvironment might affect the ability of these regimens to induce immunogenic tumour cell death, however, that important research question is outside the scope of this current study.

FLOT treatment significantly increased the percentage of OE33 and SK-GT-4 cells expressing HMGB1 (OE33: untrx 2.36 ± 0.5 vs 11.23 ± 1.8%, *p* = 0.0007 and SK-GT-4: 5.63 ± 1.2 vs 13.17 ± 2.0%, *p* = 0.004), (Fig. 1B). Conversely, MAGIC treatment significantly decreased the percentage of OE33 cells expressing HMGB1 (2.36 ± 0.5 vs 0.50 ± 0.1%, *p* = 0.03) and specifically epirubicin, a component of the MAGIC regimen, significantly decreased the percentage of SK-GT-4 cells expressing HMGB1 (5.63 ± 1.2 vs. 1.09 ± 0.5%, *p* = 0.02), (Fig. 1B) Treatment with the remaining single agent chemotherapies or CROSS CT regimen did not significantly affect the expression levels of HMGB1.

Following FLOT, CROSS CT and MAGIC chemotherapy treatment, the percentage of OE33 cells and SK-GT-4 cells expressing calreticulin did not significantly change (Fig. 1B). However, single agent treatment with carboplatin and cisplatin, which comprise the CROSS regimen and MAGIC regimen, respectively, significantly increased the percentage of OE33 cells expressing calreticulin (untrx: 5.59 ± 1.6% vs. carboplatin: 33.57 ± 1.8% *p* = 0.01, and cisplatin: 27.57 ± 1.7%, *p* = 0.03) (Fig. 1B). Additionally, following single agent 5-FU and docetaxel treatment there was a trend toward an increase in the percentage of OE33 cells expressing calreticulin (untrx: 5.59 ± 1.6% vs. 5-FU: 24.03 ± 1.6% *p* = 0.06, and docetaxel: 30.07 ± 3.3%, *p* = 0.06) (Fig. 1B).

**Fig. 2:**
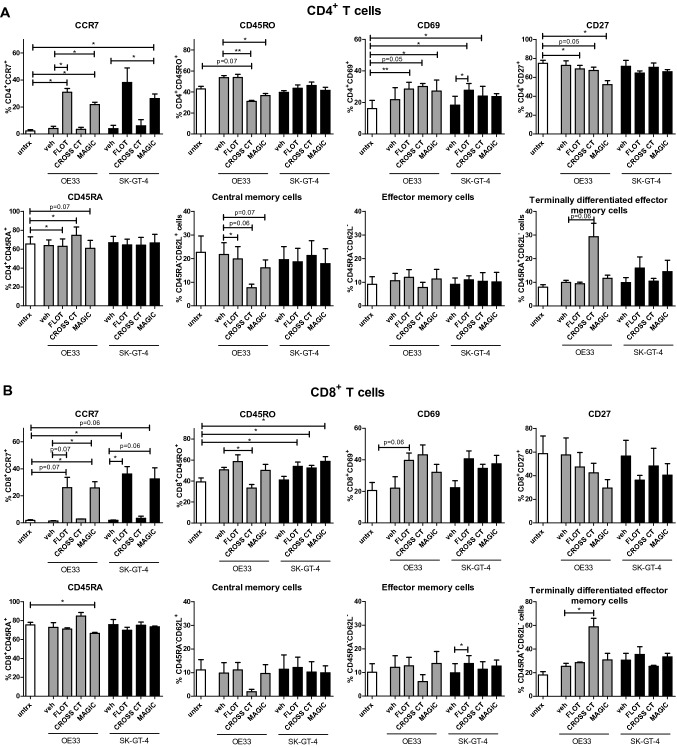
Conditioned media from FLOT and CROSS chemotherapy-treated OGJ cells increases the percentage of viable T cells expressing CD69, whereas FLOT and MAGIC decrease CD27 expression. PBMCs were activated with plate bound anti-CD3 and anti-CD28 and untreated (untrx) or concurrently treated with supernatant harvested from OE33 and SK-GT-4 cells vehicle control treated (veh) or treatment with FLOT, CROSS CT or MAGIC chemotherapy regimens for 48 h (IC_50_ dose). The effect of the supernatant on viable CD3^+^CD4^+^ (**A**) and CD3^+^CD8^+^ (**B**) T cell activation status was determined by CCR7, CD45RO, CD62L, CD45RA, CD27 and CD69 expression by flow cytometry. The percentage of viable naïve (CD45RA^+^CD62L^+^), central memory (CD45RA^−^CD62L^+^), pre-terminally differentiated effector memory (CD45RA^−^CD62L^−^) and effector memory (CD45RA^+^CD62L^−^) CD3^+^CD4^+^ (**A**) and CD3^+^CD8^+^ (**B**) cells was also determined by flow cytometry. A zombie viability dye was used to exclude dead cells from the analysis. *n* = 3 supernatants from each cell line was used to treat one healthy donor. Paired parametric *t*-test, *p < 0.05 and **p < 0.01

Single agent 5-FU and oxaliplatin (comprise the FLOT regimen) significantly increased the percentage of OE33 cells expressing MIC-A/B (untrx: 31.54 ± 11.7 vs. 5-FU: 62.75 ± 5.6%, *p* = 0.007 and oxaliplatin: 65.73 ± 5.8%, *p* = 0.004) (Fig. 1B). Similar findings were found in the SK-GT-4 cells using 5-FU and docetaxel and the FLOT combination regimen (untrx: 7.84 ± 1.7 vs. 5-FU: 17.06 ± 1.3%, *p* = 0.02, docetaxel: 21.82 ± 3.0% *p* = 0.02 and FLOT: 20.8 ± 3.5%, *p* = 0.02) (Fig. 1B). Single agent carboplatin or paclitaxel and the combination CROSS CT regimen did not significantly affect the percentage of OE33 cells expressing MIC-A/B, whereas single agent carboplatin, paclitaxel and CROSS CT regimen significantly increased the percentage of SK-GT-4 cells expressing MIC-A/B (untrx: 7.84 ± 1.7% vs. carboplatin: 25.7 ± 5.1%, *p* = 0.04, paclitaxel: 29.96 ± 4.0%, *p* = 0.007 and CROSS CT: 31.43 ± 2.8%, *p* = 0.0005) (Fig. 1B). Furthermore, single agent epirubicin significantly decreased the percentage of OE33 and SK-GT-4 cells expressing MIC-A/B (untrx: 7.84 ± 1.7% vs. epirubicin: 4.31 ± 0.9%, *p* = 0.001) Fig. 2). Whereas, cisplatin significantly increased MIC-A/B expression levels in the OE33 cells and SK-GT-4 cells (untrx: 7.84 ± 1.7% vs. cisplatin: 27.8 ± 1.0%, *p* = 0.007). MAGIC regimen only significantly increased the percentage of MIC-A/B positive cells in the OE33 cell line (untrx: 7.84 ± 1.7% vs. MAGIC: 29.82 ± 10.2%, *p* = 0.007) but not the SK-GT-4 cell line (Fig. 1B).

Treatment with single agent chemotherapies comprising the FLOT regimen as well as the combination FLOT regimen significantly increased the percentage of OE33 cells expressing HLA-DR (untrx: 1.06 ± 0.1% vs. 5-FU: 3.27 ± 0.2%, *p* = 0.0002, docetaxel: 2.60 ± 0.5%, *p* = 0.04 and FLOT: 8.7 ± 1.5%, *p* = 0.002) (Fig. 1B). Similar findings were observed in the SK-GT-4 cell line (untrx: 0.89 ± 0.1% vs. 5-FU: 3.35 ± 0.7%, *p* = 0.01, oxaliplatin: 3.6 ± 0.6%, *p* = 0.003, docetaxel: 4.17 ± 0.9%, *p* = 0.01 and FLOT: 5.0 ± 0.3%, *p* < 0.0001) (Fig. 1B). Single agent treatment with carboplatin or paclitaxel did not significantly affect the percentage of OE33 cells expressing HLA-DR, however, CROSS CT treatment significantly increased the percentage of HLA-DR^+^OE33 cells (untrx: 1.06 ± 0.1% vs. CROSS CT: 3.46 ± 0.6%, *p* = 0.006). Similar findings were observed in the SK-GT-4 cell line where single agent carboplatin and paclitaxel as well as the combination of both (CROSS CT) significantly increased the percentage of HLA-DR^+^SK-GT-4 cells (untrx: 0.89 ± 0.1% vs. carboplatin: 3.34 ± 1.0%, *p* = 0.04, paclitaxel: 5.80 ± 1.0%, *p* = 0.002 and CROSS CT: 4.11 ± 0.7%, *p* = 0.003) (Fig. 1B). Cisplatin significantly increased the percentage of OE33 and SK-GT-4 cells expressing HLA-DR whereas, epirubicin significantly increased HLA-DR expression on OE33 cells yet paradoxically decreased the percentage of SK-GT-4 cells expressing HLA-DR (OE33 untrx: 1.06 ± 0.1% vs. cisplatin: 4.43 ± 0.9%, *p* = 0.02 and epirubicin: 5.0 ± 1.07%, *p* = 0.006 and SK-GT-4: untrx: 0.89 ± 0.1% vs. cisplatin: 8.03 ± 1.7%, *p* = 0.004 and epirubicin: 0.18 ± 0.04%, *p* = 0.0008). Additionally, there was a trend toward a decrease in HLA-DR expression on OE33 cells following MAGIC treatment (1.06 ± 0.1% vs. 0.64 ± 0.1%, *p* = 0.05, respectively).

Overall, the FLOT and CROSS CT regimens demonstrated the greatest immunogenic potential via upregulation of surrogate markers of ICD upon treatment with single agent chemotherapies comprising these regimens or the combination of these chemotherapies. MAGIC demonstrated the least immunogenic potential and in particular single agent epirubicin decreased surrogate markers of ICD and may be a key contributor to the reduced ICD potential of MAGIC compared with FLOT and CROSS CT regimens.

### Conditioned media from FLOT and CROSS CT chemotherapy-treated OGJ cells alters the activation phenotype of T cells in vitro

To successfully identify immunostimulatory chemotherapies as synergistic partners to combine with immunotherapies for OGJ patients, it is important to elucidate how the chemotherapy regimens may alter the secretome from OGJ cells which may subsequently affect T cell activity. To address this, we examined the effect of in vitro conditioned media from FLOT, CROSS CT and MAGIC chemotherapy-treated OGJ cells on T cell activation. The markers used to assess CD3^+^, CD3^+^CD4^+^ and CD3^+^CD8^+^ T cell activation included: CCR7, CD45RO, CD27, CD69, CD45RA and CD62L, determined by flow cytometry (Fig. 2). The percentages of viable naïve (CD45RA^+^CD62L^+^), central memory (CD45RA^−^CD62L^+^), effector memory (CD45RA^−^CD62L^−^) and terminally differentiated effector memory (CD45RA^+^CD62L^−^) CD3^+^CD4^+^ and CD3^+^CD8^+^ cells were also determined (Maldonado et al. 2003) (Fig. 2).

It is important to note that the healthy donor PBMCs that are treated with FLOT/CROSS-treated OAC cell medium are not autologous and this might lead to unspecific immune activation as a result of potentially different HLA classes and non-self-epitopes. Therefore, comparing the untreated OAC cell conditioned medium with the FLOT/CROSS CT OAC cell conditioned medium acts as a control that takes into account any unspecific immune activation that is not attributed to the FLOT or CROSS CT altered tumour cell secretome.

FLOT and MAGIC OE33 conditioned media increased the percentage of CCR7-expressing CD3^+^CD4^+^ T cells (untreated OE33: 4.08 ± 1.7 vs. FLOT OE33: 31 ± 2.7 and MAGIC OE33: 21.83 ± 1.7%, respectively, *p* = 0.01 and *p* = 0.03, respectively), (Fig. 2A). CROSS CT and MAGIC OE33 conditioned media decreased the percentage of CD3^+^CD4^+^ T cells expressing CD45RO marker of T cell memory (OE33 untreated: 53.70 ± 2.0 vs. CROSS CT OE33: 31 ± 1.0 and MAGIC OE33: 36.57 ± 2.0%, respectively, *p* = 0.01 and *p* = 0.04, respectively), (Fig. 2A). FLOT and MAGIC OE33 conditioned media increased the percentage of CD69-expressing CD3^+^CD4^+^ T cells (untreated: 16.14 ± 5.2 vs. FLOT OE33: 28.5 ± 4.3 and MAGIC OE33: 27.27 ± 7.0%, respectively, *p* = 0.008 and *p* = 0.04, respectively), (Fig. 2A). There was a trend toward CROSS CT OE33 conditioned media increasing the percentage of CD69-expressing CD3^+^CD4^+^ T cells (untreated: 16.14 ± 5.2 vs. CROSS CT OE33: 30.19 ± 1.9%, respectively, *p* = 0.05), (Fig. 2A). Similarly, FLOT and CROSS CT SK-GT-4 conditioned media increased the percentage of CD69-expressing CD3^+^CD4^+^ cells (untreated: 16.14 ± 5.2, FLOT SK-GT-4: 27.77 ± 4.3 and CROSS CT SK-GT-4: 24.03 ± 6.2%, respectively, *p* = 0.01 and *p* = 0.02, respectively), (Fig. 2A). FLOT and MAGIC OE33 conditioned media decreased the percentage of CD27-expressing CD3^+^CD4^+^ T cells (untreated: 74.83 ± 3.1 vs. FLOT OE33: 68.83 ± 3.32 and MAGIC OE33: 52.17 ± 4.3%, respectively, *p* = 0.03 and *p* = 0.04, respectively) (Fig. 2A). There was a trend toward CROSS CT significantly decreasing the percentage of CD27-expressing CD3^+^CD4^+^ T cells (untreated: 74.83 ± 3.1 vs. CROSS CT OE33: 67.23 ± 3.5%, respectively *p* = 0.0536), (Fig. 2A). CROSS CT OE33 conditioned media decreased the percentage of CD45RO^+^ CD3^+^CD8^+^ T cells (untreated OE33: 50.63 ± 2.3 vs. CROSS CT OE33: 33.27 ± 3.4%, respectively, *p* = 0.03), (Fig. 2B). In contrast, FLOT, CROSS CT and MAGIC SK-GT-4 conditioned media increased the percentage of CD45RO^+^ CD3^+^CD8^+^ cells (untreated: 39.17 ± 3.9 vs. FLOT SK-GT-4: 53.9 ± 4.0, CROSS CT SK-GT-4: 52.47 ± 2.4 and MAGIC SK-GT-4: 58.6 ± 4.6%, respectively, *p* = 0.04, *p* = 0.01 and *p* = 0.01, respectively), (Fig. 2B).

In addition, FLOT OE33 conditioned media significantly decreased the percentage of central memory CD3^+^CD4^+^ cells (OE33 untreated: 21.73 ± 4.9 vs. FLOT OE33: 19.9 ± 5.2%, respectively, *p* = 0.02), (Fig. 2A). FLOT SK-GT-4 conditioned media significantly increased the percentage of effector memory CD3^+^CD8^+^ cells (SK-GT-4 untreated: 9.49 ± 3.9 vs. FLOT SK-GT-4: 13.82 ± 3.3%, respectively, *p* = 0.04), (Fig. 2B). Furthermore, CROSS CT-treated OE33 conditioned media significantly increased the percentage of terminally differentiated effector memory CD3^+^CD8^+^ cells (OE33 untreated: 25.33 ± 2.5 vs. CROSS CT OE33: 58.87 ± 7.2%, respectively, *p* = 0.02), (Fig. 2B).

Overall, the conditioned media from OGJ cells treated with combination chemotherapy regimens significantly altered the activation status of T cells. Specifically, FLOT and MAGIC regimens significantly enhanced frequencies of CCR7-expressing T cells, whereas FLOT and CROSS CT treatment increased frequencies of CD69-expressing T cells yet decreased frequencies of their CD27-expressing counterparts. In addition, FLOT and CROSS CT chemotherapy regimens significantly decreased the frequencies of central memory T cells, whilst increasing the percentages of effector memory T cells.

**Fig. 3:**
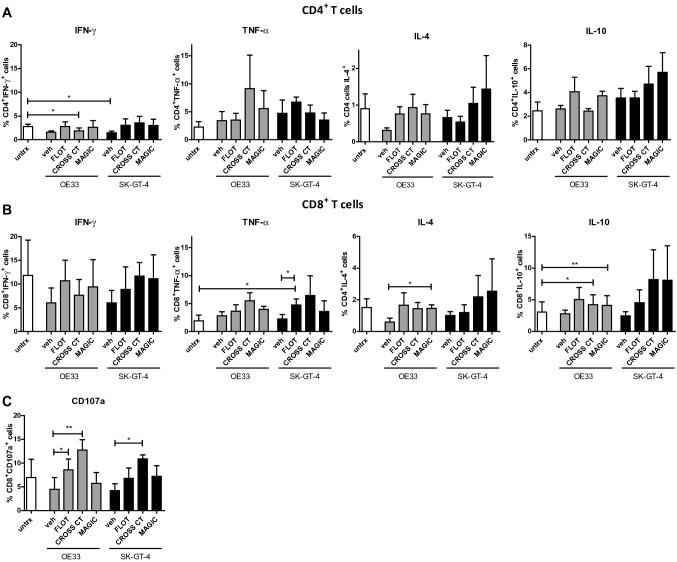
Conditioned media from FLOT and CROSS chemotherapy-treated OGJ cells increases the cytotoxic potential of CD8^+^ T cells in vitro. Activated PBMCs were untreated or concurrently treated with supernatant harvested from vehicle control treated (veh) OE33 and SK-GT-4 cells or following 48 h treatment with FLOT, CROSS CT or MAGIC chemotherapy regimens (IC_50_ dose). The effect of the supernatant on viable CD3^+^CD4^+^ (**A**) and CD3^+^CD8^+^ (**B**) T cell cytokine production was determined by assessing the expression of IFN-γ, TNF-α, IL-10, IL-4 and IL-17A by intracellular flow cytometry. **C** The effect of the supernatant on viable CD3^+^CD8^+^ T cell cytotoxicity was determined by assessing the expression of CD107a expression by flow cytometry. A zombie viability dye was used to exclude dead cells from the analysis and *n* = 3 biological replicates of supernatants from each cell line was used to treat one healthy donor. Paired parametric *t*-test, *p < 0.05 and **p < 0.01

The immune contexture of solid tumours is shaped by the cell types, profiles, polarisation and modulation of immune cells within the TME and ultimately influences the resulting anti-tumour response and ICI efficacy. T cells play a pivotal role in OGJ development and progression, depending on the cytokine profile, and may suppress or enhance anti-tumour immunity through T_H2_-like responses T_H1_-like responses, respectively (Sandick et al. 2003). Herein, we examined the effects of soluble factors secreted by OGJ cells following FLOT, CROSS CT and MAGIC on T cell cytokine profiles and CD8^+^ T cell degranulation to elucidate the effects of pre-treated TME on T cell function. The secretion of T_H1_-like cytokines including IFN-γ and TNF-α, T_H2_-like cytokines including IL-4 and IL-10 and the T_H17_-like cytokine IL-17A were assessed by intracellular flow cytometry in CD3^+^, CD3^+^CD4^+^ and CD3^+^CD8^+^ cells (Fig. 3). CROSS CT and MAGIC OE33 conditioned media appeared to enhance a T_H2_-like phenotype in T cells, (Fig. 3). CROSS CT OE33 conditioned media enhanced a T_H2_-like/regulatory-like phenotype in T cells demonstrated by a decrease in IFN-γ-producing CD3^+^CD4^+^ T cells (untreated: 2.78 ± 0.5 vs. CROSS CT OE33: 1.83 ± 0.6%, respectively, *p* = 0.03), (Fig. 3A) and an increase in IL-10-producing CD3^+^CD8^+^ T cells (untreated: 3.07 ± 1.6 vs. CROSS CT OE33: 4.24 ± 1.6%, respectively, *p* = 0.01), (Fig. 3B). Similarly, MAGIC OE33 conditioned media enhanced a T_H2_-like phenotype in T cells demonstrated by an increase in IL-4-producing CD3^+^CD8^+^ T cells (untreated OE33: 0.60 ± 0.2 vs. MAGIC OE33: 1.45 ± 0.2%, respectively, *p* = 0.02) and IL-10-producing CD3^+^CD8^+^ T cells (untreated: 3.07 ± 1.6 vs. MAGIC OE33: 4.13 ± 1.5%, respectively, *p* = 0.007), (Fig. 3B). In contrast, FLOT SK-GT-4 conditioned media promoted a more T_H1_-like phenotype in T cells demonstrated by a significant increase in TNF-α-producing CD3^+^CD8^+^ T cells (untreated SK-GT-4: 2.25 ± 0.8 vs. FLOT SK-GT-4: 4.75 ± 1.1%, respectively, *p* = 0.02), (Fig. 3B). Additionally, we also examined the effect of FLOT, CROSS CT and MAGIC OGJ cell conditioned media on CD8^+^ T cell cytotoxic potential by CD107a expression (Fig. 3C). The FLOT and CROSS CT OE33 conditioned media significantly increased the percentage of CD107a^+^CD3^+^CD8^+^ T cells (OE33 untreated: 4.47 ± 2.5 vs. OE33 FLOT: 8.58 ± 2.3% and OE33 CROSS CT: 12.74 ± 2.2%, respectively, *p* = 0.004 and *p* = 0.01, respectively), (Fig. 3C). Similarly, the CROSS CT SK-GT-4 conditioned media significantly increased the percentage of viable CD107a^+^CD3^+^CD8^+^ T cells (SK-GT-4 untreated: 4.2 ± 1.5 vs. SK-GT-4 CROSS CT: 10.87 ± 0.8%, respectively, *p* = 0.04), (Fig. 3C)

Overall, FLOT and CROSS CT conditioned media from OE33 cells enhanced T_H2_-like phenotype. In contrast, FLOT conditioned media from SK-GT-4 cells enhanced a T_H1_-like phenotype. CROSS CT conditioned media from both OE33 and SK-GT-4 cells enhanced the cytotoxic potential of T cells, whereas only the FLOT conditioned media from OE33 cells enhanced CD107a expression. However, there was no significant effect on granzyme B production or perforin expression by CD8^+^ T cells following treatment with vehicle or chemotherapy-treated OGJ cell conditioned media (data not shown).

### Tumour conditioned media (TCM) from OGJ tumour biopsies post-FLOT and post-CROSS CRT treatment significantly alters the activation profile of T cells ex vivo

In light of the in vitro results using conditioned media from OGJ cell lines treated with FLOT or CROSS CT regimens, which reflected the secreted factors from just epithelial cells, we next assessed tumour biopsy conditioned media (TCM) from post-FLOT and post-CROSS CRT treated patients. The TCM facilitated simulation of the entire tumour microenvironment comprising the secreted soluble factors from all of the cells within the tumour microenvironment. Recognising and appreciating the inter-patient heterogeneous nature of the tumour microenvironment, we used TCM generated from 11 patients that received either FLOT (*n* = 5) or CROSS CRT neoadjuvant therapy to test how the chemotherapy-altered tumour microenvironment affects T cell activation status. Due to the changes in the standard of care for treating OGJ patients in St James’s Hospital in Dublin, Ireland where this study was performed only FLOT and CROSS CRT are given to OGJ patients as of 2018. This clinical decision was based on findings from the clinical trial FLOT4 trial which demonstrated that FLOT improved overall survival more significantly than MAGIC in OGJ patients (50 vs. 35 months) (Al-Batran et al. 2019). Therefore, this study only included prospectively collected fresh tumour tissue samples from OGJ patients who were receiving FLOT or CROSS CRT.

**Fig. 4:**
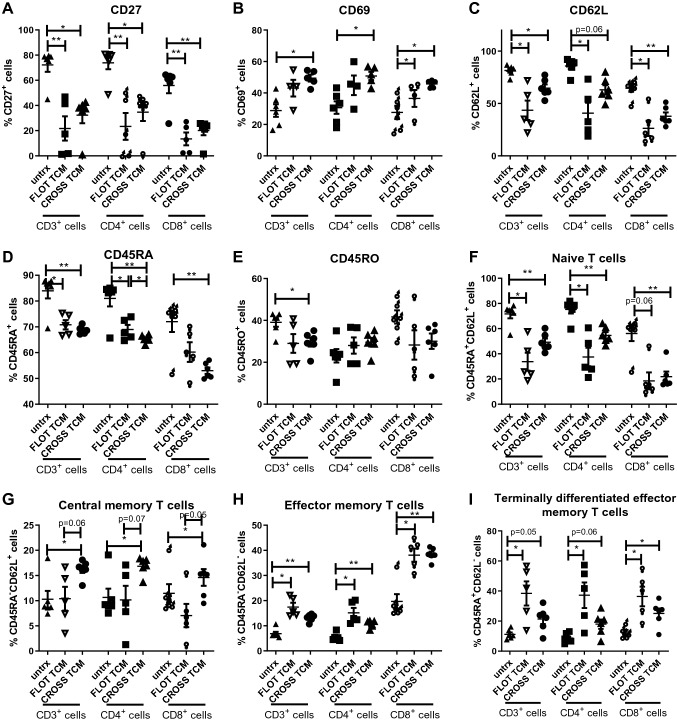
Conditioned media generated from OGJ patient-derived tumour at time points post-FLOT and post-CROSS treatment significantly increases the frequencies of CD69^+^, central memory and effector memory T cells, yet decreases the percentage of CD27-expressing T cells ex vivo*.* Healthy donor PBMCs were activated with plate bound anti-CD3 and anti-CD28 in the absence or presence of post-FLOT (*n* = 5) and post-CROSS (*n* = 6) conditioned media for 48 h. CD3^+^, CD3^+^CD4^+^ and CD3^+^CD8^+^ cells were assessed for CD27 (**A**), CD69 (**B**), CD62L (**C**), CD45RA (**D**) and CD45RO (E) expression by flow cytometry. The percentages of naïve (CD45RA^+^CD62L^+^) (**F**), central memory (CD45RA^−^CD62L^+^) (**G**), pre-terminally differentiated effector memory (CD45RA^−^CD62L^−^) (**H**) and effector memory (CD45RA^+^CD62L^−^) (**I**) cells were determined by flow cytometry. Paired non-parametric *t*-test. *p < 0.05, **p < 0.01

Healthy donor PBMCs were activated in the presence or absence of post-FLOT or post-CROSS CRT TCM. The ex vivo findings validated the results of the in vitro experiments demonstrating that post-FLOT and post-CROSS CRT conditioned media significantly decreased the percentage of CD3^+^CD27^+^ cells (72.23 ± 5.5 vs. 21.72 ± 9.6 and 32.28 ± 6.4%, *p* = 0.003 and *p* = 0.02, respectively), (Fig. 4A). Similar findings were found within the CD4^+^ and CD8^+^ T cell compartments. In addition, the post-FLOT and post-CROSS CRT conditioned media significantly increased the percentage of CD3^+^CD8^+^CD69^+^ cells compared with untreated cells (

27.62 ± 4.2 vs. 43.55 ± 5.4% and 45.76 ± 1.1%, *p* = 0.04 and *p* = 0.01, respectively), (Fig. 4B). Similar findings were observed using the post-CROSS CRT conditioned media only within the CD4^+^ T cell compartment. The percentage of CD62L expressing CD3^+^ cells were significantly decreased in the post-FLOT and post-CROSS conditioned media compared with untreated cells (81.52 ± 2.0 vs. 43.52 ± 9.2% and 64.67 ± 3.6%, *p* = 0.02 and *p* = 0.01, respectively), (Fig. 4C). Similar findings were observed in the CD4 and CD8 cell compartment (Fig. 4A). Frequencies of CD45RA^+^ T cells were significantly decreased within the CD3^+^ population and within the CD4^+^ and CD8^+^ subsets following treatment with either post-FLOT and post-CROSS CRT TCM compared with untreated cells (83.97 ± 2.9 vs. 70.82 ± 1.8% and 68.78 ± 0.6%, *p* = 0.03 and *p* = 0.003, respectively), (Fig. 4D). Similar trends were observed in the CD4 and CD8 T cell compartments. Furthermore, the percentage of CD3^+^CD45RO^+^ T cells were significantly decreased following treatment with the post-CROSS CRT TCM compared with untreated cells (39.05 ± 2.1 vs. 29.6 ± 2.1%, *p* = 0.02), (Fig. 4E). No significant changes were observed for changes in the percentages of CD4^+^ or CD8^+^ T cells expressing CD45RO following treatment with either the post-FLOT or post-CROSS CRT TCM compared with untreated cells.

Following treatment with either the post-FLOT or post-CROSS CRT TCM the percentages of naïve CD3^+^, CD3 + CD4 + and CD3^+^CD8^+^ T cells were significantly decreased, characterised by CD45RA and CD62L co-expression (Fig. 4F). Additionally, treatment with post-CROSS CRT TCM significantly increased the percentage of central memory T cells within the CD3^+^, CD3 + CD4 + and CD3^+^CD8^+^ T cell compartment (Fig. 4G). Effector memory CD3^+^ cells were increased by post-FLOT and post-CROSS CRT TCM, characterised by CD45RA^−^CD62L^−^ (6.9 ± 0.8 vs. 17.42 ± 1.7% and 13.22 ± 0.6%, *p* = 0.01 and *p* = 0.001), (Fig. 4H). Similar increases in effector memory T cells were specifically observed in the CD3^+^CD4^+^ and CD3^+^CD8^+^ compartment by post-FLOT and post-CROSS CRT TCM treatment, characterised by CD45RA^−^CD62L^−^. Finally, the percentage of terminally differentiated effector memory CD3^+^, CD3^+^CD4^+^ and CD3^+^CD8^+^ T cells were significantly increased following treatment with post-FLOT and post-CROSS CRT TCM, characterised by CD45RA^+^CD62L^−^(Fig. 4I).

Overall, we observed that PBMCs activated in the presence of conditioned media generated from OGJ patient-derived tumour biopsies at time-points post-FLOT and post-CROSS CRT treatment decreased CD27 and increased CD69 expression. Additionally, significant decreases in the frequencies of naïve T cells and subsequent increases in the frequencies of T cells displaying phenotypes representative of central memory, effector memory and terminally differentiated effector memory T cells were observed.

### Significantly lower frequencies of CD27-expressing tumour-infiltrating T cells were detected post-FLOT chemotherapy and post-CROSS chemoradiotherapy regimens

**Fig. 5:**
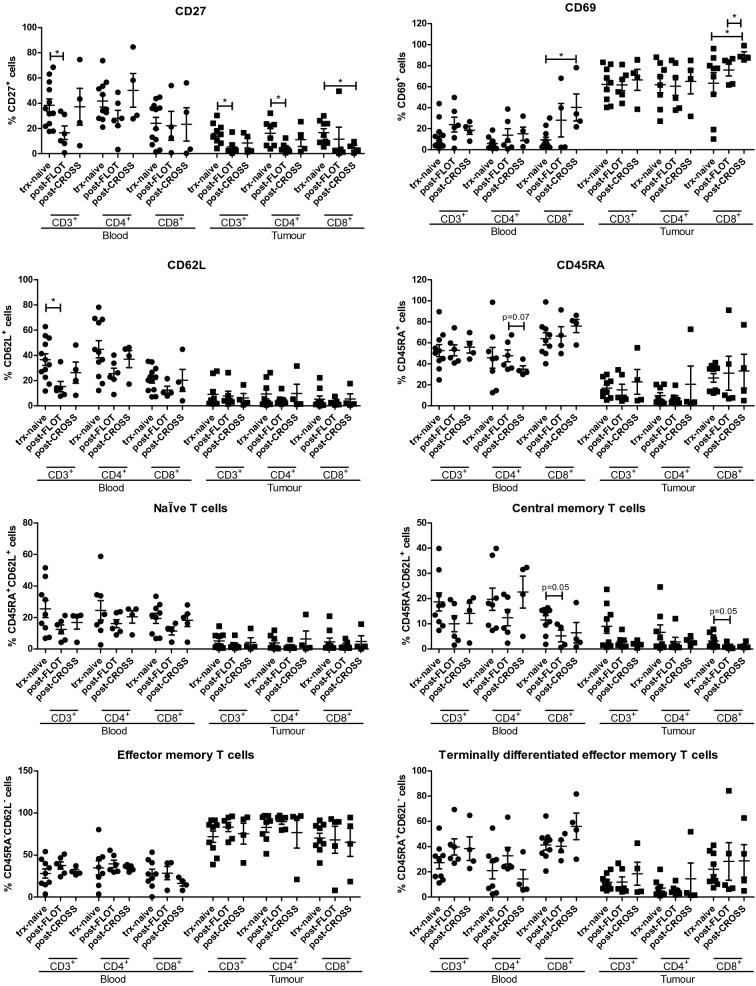
Post-FLOT chemotherapy and post-CROSS chemoradiotherapy the percentage of tumour-infiltrating T cells expressing CD27 is significantly lower whereas the percentage of tumour-infiltrating T cells expressing CD69 is significantly higher post-CROSS in OGJ patients. CD3^+^, CD3^+^CD4^+^ and CD3^+^CD8^+^ cells in circulating peripheral blood and infiltrating tumour tissue of OGJ patients in the treatment-naïve setting and post-treatment setting at surgical resection was assessed for CD27, CD69, CD62L and CD45RA expression. The percentage of naïve (CD45RA^+^CD62L^+^), central memory (CD45RA^−^CD62L^+^), effector memory (CD45RA^−^CD62L^−^) and terminally differentiated effector memory (CD45RA^+^CD62L^−^) cells was also determined by flow cytometry. (Blood: treatment-naïve: *n* = 12, post-FLOT: *n* = 5 and post-CROSS CRT: *n* = 5. Tumour: treatment-naïve: *n* = 10, post-FLOT: *n* = 5 and post-CROSS CRT: *n* = 5). Mann–Whitney test, *p < 0.05

To provide further validation of our in vitro and ex vivo data showing that FLOT and CROSS CT treatment of OGJ tumours can significantly alter the CD27 and CD69 expression and the differentiation status of T cells, we profiled the activation status of T cells in the peripheral blood and tumour of a cohort of OGJ patients in the treatment-naïve, post-FLOT and post-CROSS CRT clinical setting (Fig. 5).

Indeed, we found that the percentages of CD3^+^CD27^+^ cells in peripheral circulation were significantly lower post-FLOT chemotherapy compared to the treatment-naïve setting (38.38 ± 5.1 vs. 16.62 ± 5.4%, *p* = 0.02), (Fig. 5.). Similarly, the percentages of tumour-infiltrating CD3^+^CD27^+^ cells were significantly lower post-FLOT chemotherapy compared to the treatment-naïve setting (16.1 ± 2.7 vs. 5.41 ± 2.6%, *p* = 0.01), (Fig. 5). This was also observed in the CD4^+^ T cell compartment, with a decrease in the percentage of tumour-infiltrating CD3^+^CD4^+^CD27^+^ post-FLOT chemotherapy compared with the treatment-naïve tissue (16.22 ± 3.6 vs. 4.64 ± 1.8%, *p* = 0.03), (Fig. 5). Similarly, the frequency of tumour-infiltrating CD3^+^CD8^+^CD27^+^ T cells were significantly lower post-CROSS chemoradiation compared with the treatment-naïve setting (16.8 ± 3.1 vs. 4.64 ± 1.9%, *p* = 0.03), (Fig. 5).

In line with our in vitro and ex vivo data, the percentages of circulating CD3^+^CD8^+^CD69^+^ cells were significantly higher post-CROSS CRT treatment compared with treatment-naïve (9.34 ± 2.6 vs. 40.25 ± 12.8%, *p* = 0.01), (Fig. 5). Similar findings were observed in the tumour tissue, wherein post-CROSS CRT the percentages of tumour-infiltrating CD3^+^CD8^+^CD69^+^ T cells were significantly higher compared with treatment-naïve tumour tissue (63.3 ± 10.0 vs. 90.08 ± 3.0%, *p* = 0.04), (Fig. 5). The frequencies of circulating peripheral blood CD3^+^CD62L^+^ T cells were significantly lower post-FLOT chemotherapy compared with treatment-naive (36.62 ± 4.7 vs. 15.34 ± 4.1%, *p* = 0.01), (Fig. 5). Additionally, there was a trend toward lower frequencies of peripheral blood circulating central memory CD3^+^CD8^+^ T cells post-FLOT compared with treatment-naïve (11.55 ± 1.7 vs. 5.22 ± 2.4%, *p* = 0.05), (Fig. 5). A similar trend towards significance was also observed in the tumour tissue where a decrease in tumour-infiltrating central memory CD3^+^CD8^+^ T cells post-FLOT was observed compared with treatment-naive (3.23 ± 0.9 vs. 1.33 ± 0.6%, *p* = 0.06), (Fig. 5).

### Frequencies of CD69^+^ T cells following 48 h culture with post-FLOT/CROSS CRT conditioned media negatively correlated with levels of soluble pro-angiogenic and immunosuppressive factors PIGF and VEGFA present in the TCM

**Fig. 6:**
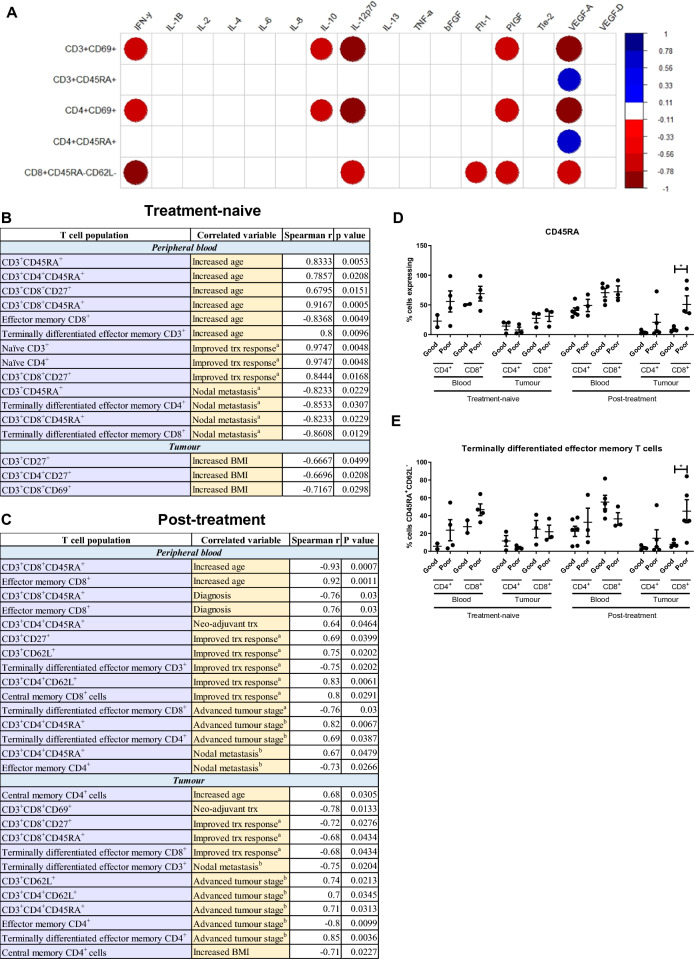
Frequencies of CD69^+^ T cells following 48 h culture with post-FLOT/CROSS CRT conditioned media negatively correlated with levels of soluble pro-angiogenic and immunosuppressive factors PIGF and VEGFA present in the TCM. (**A)** Corogram demonstrating the correlation values between T cell activation status following 24 h treatment with post-FLOT or post-CROSS CRT conditioned media with the levels of pro-inflammatory and pro-angiogenic analytes in the TCM. Only significant correlations shown. Spearman *r* 0.4–0.59 moderate, 0.6–0.79 strong and 0.8–1 very strong. Blue and red refer to positive and negative correlations, respectively. *p < 0.05, **p < 0.01. Correlation values for peripheral blood and tumour-infiltrating T cell activation status with clinical characteristics in the treatment-naïve tissue (**B**) and post-treatment tissue (**C**). Only significant correlations shown. Spearman *r* 0.4–0.59 moderate, 0.6–0.79 strong and 0.8–1 very strong. CROSS CRT = 0, FLOT = 1, OGJ = 0, OGJ = 1, female = 0, male = 1. *p < 0.05, **p < 0.01. **D–E** CD3^+^CD4^+^ and CD3^+^CD8^+^ cells in circulating peripheral blood and infiltrating tumour tissue of OGJ patients in the treatment-naïve setting and post-treatment setting at surgical resection were assessed for CD27, CD69, CD62L and CD45RA expression based on pathologic response to neoadjuvant treatment. The percentage of naïve (CD45RA^+^CD62L^+^), central memory (CD45RA^−^CD62L^+^), effector memory (CD45RA^−^CD62L^−^) and terminally differentiated effector memory (CD45RA^+^CD62L^−^) cells was also determined by flow cytometry and graphed based on pathologic response to neoadjuvant treatment. Good pathologic response: tumour regression grade 1–2 and poor pathologic response: tumour regression grade: 3–5. Only significant data shown. Treatment-naïve blood: good responders (*n* = 5) and poor responders (*n* = 4), treatment-naïve tumour tissue: good responders (*n* = 3) and poor responders (*n* = 3). Post-treatment blood: good responders (*n* = 6) and poor responders (*n* = 3), post-treatment tumour tissue: good responders (*n* = 4) and poor responders (*n* = 5). Mann–Whitney test, *p < 0.05

Given the high prevalence of treatment failure and lack of clinical biomarkers to predict response to neo-adjuvant CT and CRT, we sought to investigate if the frequency of activated T cells in peripheral blood circulation or infiltrating tumour tissue biopsies correlated with a better prognosis or subsequent treatment response in the treatment-naïve cohort of this study (Fig. 6B) and the post-treatment cohort of patients (Fig. 6C). The frequencies of circulating peripheral blood naïve CD3^+^ cells (*r* = 0.97) and CD4^+^ T cells (*r* = 0.97) very strongly correlated with a subsequent better treatment response (*p* = 0.005 and *p* = 0.005, respectively), (Fig. 6B). Additionally, the frequencies of circulating peripheral blood CD3^+^CD8^+^CD27^+^ (*r* = 0.84) very strongly positively correlated with a subsequent better treatment response (*p* = 0.02) (Fig. 6B). The frequency of circulating peripheral blood terminally differentiated effector memory CD8^+^ cells (*r* = − 0.86, *p* = 0.01) and CD4^+^ cells (*r* = − 0.85, *p* = 0.03) negatively correlated with lymph node metastasis in the treatment-naïve setting (Fig. 6B).

The frequency of peripheral blood circulating CD3^+^CD27^+^ T cells strongly correlated (*r* = 0.69) with a better treatment response in the post-treatment setting (*p* = 0.04), (Fig. 6C). There was a very strong positive correlation (*r* = 0.80) with the frequency of peripheral blood circulating central memory CD8^+^ cells with a better treatment response in the post-treatment setting (Fig. 6C).

In addition, T cell activation status following 48 h treatment with post-FLOT or post-CROSS CRT conditioned media was also correlated with the levels of soluble pro-inflammatory and pro-angiogenic analytes that were present in the TCM. The percentage of CD4^+^CD69^+^ T cells and effector memory CD8^+^ T cells negatively correlated with the levels of pro-angiogenic factors PIGF and VEGFA present in the conditioned media (CD4^+^CD69^+^ T cells: *r* = − 0.76 (*p* = 0.01) and − 0.89 (*p* = 0.0005), respectively, and effector memory CD8^+^ T cells: *r* = − 0.72 (*p* = 0.01) and − 0.77 (*p* = 0.009), respectively) (Fig. 6A).

Overall, T cell activation status of circulating and tumour-infiltrating T cells correlated with response to treatment and adverse tumour biology as well as levels of immunosuppressive and pro-angiogenic PIGF and VEGFA present within the TME. The expression of T cell activation markers and frequency of T cell subsets were also assessed based on good versus poor pathologic response to neoadjuvant treatment. OGJ patients who had a poor pathologic response to treatment had significantly higher levels of tumour-infiltrating CD3^+^CD8^+^CD45RA^+^ T cells (50.82 ± 14.7 vs. 8.47 ± 2.0%, *p* = 0.03), (Fig. 6D) and terminally differentiated effector memory T cells (45.05 ± 12.9 vs. 7.76 ± 1.8%, *p* = 0.03) compared with good responders (Fig. 6E).

### The chemotherapy-treated OE33 tumour cell secretome enhances lymphocyte-mediated killing of OE33 cells ex vivo

**Fig. 7:**
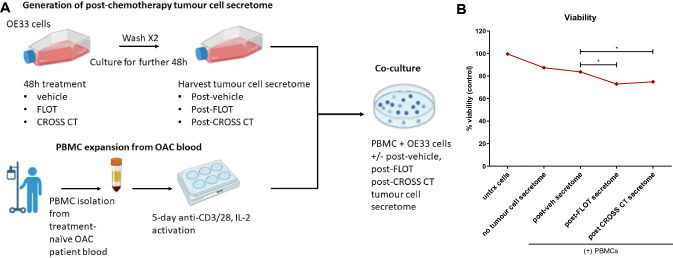
Post-FLOT- and post-CROSS CT-treated OE33 conditioned media enhances OGJ lymphocyte-mediated killing of OE33 cells. OE33 cells were untreated (untrx) or treated with vehicle control (veh)-, FLOT- or CROSS CT-treated OE33 conditioned media (CM) for 48 h. OGJ PBMCs were also co-cultured with OE33s in an effector:target (E:T) ratio of 5:1 (50,000:10,000) for 48 h. PBMCs were isolated from treatment-naïve OGJ patients (*n* = 6) and were activated for 5 days prior to the co-culture with OGJ cells using anti-CD3/28 and IL-2. A CCK8 assay was used to determine the viability of OE33 cells (*n* = 6). Mann–Whitney *t*-test, *n* = 2 technical replicates, *p < 0.05

Overall, our in vitro and ex vivo findings suggest that chemotherapy regimens may enhance the immunogenicity of OGJ cells and enhance T cell activation to ultimately improve anti-tumour immunity and elimination of the tumour. Therefore, as a proof-of-principle experiment to test this hypothesis we carried out a cytolysis assay to determine if the post-FLOT and post-CROSS CT altered OE33 tumour cell secretome might alter the killing capacity of lymphocytes isolated from treatment-naïve OGJ patient blood. OE33 cells were treated for 48 h with chemotherapy regimens, washed twice and the OE33 conditioned media was collected 48 h later. OGJ treatment-naïve PBMCs were activated with anti-CD3/28 and IL-2 ex vivo and co-cultured with OE33 cells in the presence of untreated OE33 conditioned media or post-FLOT- and post-CROSS CT-treated OE33 tumour cell conditioned media to determine if the lymphocyte mediated-killing of OE33 cells would be enhanced by the secretome of OE33 cells that had been pre-treated for 48 h with standard of care chemotherapy regimens (Fig. 7).

The post-FLOT OE33 tumour cell secretome significantly enhanced OGJ lymphocyte-mediated killing of OE33 cells ex vivo compared with the post-vehicle OE33 tumour cell secretome (5:1 E:T: 72.98 ± 11.5 vs 83.74 ± 11.5%, *p* = 0.03) (Fig. 7). Similarly, the post-CROSS CT OE33 tumour cell secretome significantly enhanced OGJ lymphocyte-mediated killing of OE33 cells ex vivo compared with the post-veh OE33 tumour cell secretome (5:1 E:T: 74.9 ± 9.7 vs. 83.74 ± 11.5%, *p* = 0.03) (Fig. 7).

Overall, these findings suggest that the FLOT and CROSS CT regimens alter the OE33 tumour cell secretome which directly enhances OGJ effector lymphocyte mediated killing of OE33 cells ex vivo.

## Discussion

We sought to investigate the immunostimulatory and immunoinhibitory properties of clinically-relevant chemotherapies and combination chemotherapy regimens in the context of OGJ. Combining immunostimulatory chemotherapies with immunotherapies is an attractive approach for enhancing the efficacy of ICIs and broadening the benefit for a greater spectrum of patients. Zitvogel et al*.* recently demonstrated that immunostimulatory chemotherapies, specifically carboplatin, induce ICD in lung cancer cells and sensitises PD-1 resistant murine lung tumours to PD-1 blockade (Pfirschke et al. 2016a). This study highlights the importance of fully elucidating the immunogenic effects of clinically relevant chemotherapy regimens to help guide and inform the rational design of combination chemo-immunotherapy regimens for OGJ patients, with the goal of enhancing response rates and sensitising ICI-resistant tumours to immune checkpoint blockade. In addition, results from the recent CheckMate 649 trial further support the rationale to combine ICIs with chemotherapy to boost responses. This phase III trial demonstrated that combining nivolumab with chemotherapy significantly improved clinical overall survival compared with chemotherapy alone (14.4 months vs. 11.1 months, respectively) and reduced the risk of death by 29% (Moehler et al. 2018).

Here, the effects of single agent chemotherapies and their combination as part of the FLOT, CROSS CT and MAGIC chemotherapy regimens on immunogenicity of OGJ tumours were assessed through expression of HMGB1, CRT, MIC-A/B and HLA-DR. Overall, the FLOT and CROSS CT regimens upregulated immunogenic markers on viable OGJ cells whereas, MAGIC decreased immunogenic markers on viable OGJ cells in vitro. Similar results were observed when the expression of these markers were assessed on dead OGJ cells (data not shown), suggesting that FLOT and CROSS CT regimens induce ICD to a greater extent that the MAGIC regimen highlighting FLOT and CROSS CT regimens as more appropriate synergistic partners to combine with ICIs in OGJ. Similar results were observed with single agent chemotherapies on ICD, demonstrating that each chemotherapy that forms the FLOT or CROSS CT regimen enhanced the immunogenicity of OGJ cells via increased expression of at least one surrogate marker of ICD on viable and dead OGJ cells.

The superiority of oxaliplatin in comparison to cisplatin regarding the initiation of ICD has also been shown in other cancer types as well, supporting the findings from our study (Smyth et al. 2017; Morgan et al. 2020)*.* In addition, considering that the findings from the FLOT4 trial demonstrated that FLOT was superior than MAGIC for treating OGJ patients, increasing the progression free survival in a cohort of OGJ patients (55 versus 35 months), this might suggest that the FLOT regimen has superior anti-cancer immunomodulatory effects than the MAGIC regimen, especially considering the critical importance of the immune system in eradicating cancer. The findings from this study support that the FLOT regimen has a greater ability to induce immunogenic tumour cell death in comparison to the MAGIC regimen. Another study highlighted that the effectiveness of anthracyclines in OGJ is debateable, as a similar efficacy as with the MAGIC regimen was observed in patients receiving a platinum- and fluoropyrimidine-based two-drug combination. Collectively, our findings and the findings from other complementary studies indicate that across many tumour types platinum compounds and taxanes are the preferred chemotherapy partners for PD-1/PD-L1 ICIs, highlighting that these observations are translatable to other malignancies and might be a pan-cancer phenomenon.

An important point to consider is the effect of different tumour microenvironments on the ability of these regimens to induce immunogenic tumour cell death, which although is outside the scope of this current study is of great importance in the OGJ setting. Certainly, several studies in other cancer types, which was expertly reviewed by Palma and Lewis (2013), have made considerable strides and advancements in our understanding of how the tumour microenvironment alters the ability of chemotherapies to induce immunogenic tumour cell death. Overall, these studies have shown that different chemotherapeutic agents may induce distinct responses in different cell types such as macrophages, which can either enhance or antagonise the ability of the drugs to induce immunogenic tumour cell death, which is possibly mediated in a tumour-type dependent fashion.

However, we observed that epirubicin which comprises the MAGIC regimen decreased HMGB1, MIC-A/B and HLA-DR on OGJ cells, whereas, 5-FU and cisplatin (the other chemotherapies that comprise the MAGIC regimen) increased these markers of ICD. The key difference between the MAGIC regimen and the FLOT and CROSS regimens is the substitution of a taxane for the anthracycline epirubicin. This particular type of anthracycline has a distinct mechanism of action whereby it forms a complex with DNA by intercalation of its planar rings between nucleotide base pairs, with consequent inhibition of nucleic acid (DNA and RNA) and protein synthesis. Such intercalation triggers DNA cleavage and inhibition of topoisomerase II, resulting in cytocidal activity (Conte et al. 2000; Martin 2016). Little is known in the literature about the effect of epirubicin on ICD or whether the type of DNA damage or genotoxic stress induced by epirubicin is immunogenic or not, however, these findings suggest that they type of damage induced by epirubicin within the cancer cell may result in immunogenically silent tumour cell death in these cell lines. These results are complementary to findings from the FLOT4 clinical trial which demonstrated that FLOT improved overall survival more significantly than MAGIC in OGJ patients (50 vs. 35 months) (Al-Batran et al. 2019) and offer an immune-based explanation as to why FLOT may be a superior anti-cancer agent than MAGIC. Based on the findings from this study FLOT-based chemotherapies appear to engage the immune system more substantially than MAGIC-based chemotherapies demonstrated by a more substantial increase in DAMPs on the surface of OGJ cells following treatment.

HMGB1 binds toll-like receptor (TLR)-4 on the surface of antigen presenting cells, stimulating their activation and maturation (Bracci et al. 2014). It has been reported that docetaxel [lung adenocarcinoma (Pan et al. 2014)], oxaliplatin [colorectal cancer (Wang et al. 2018) and lung carcinoma (Haruna et al. 2020)] and 5-FU [colon carcinoma cells (Cottone et al. 2015)] all induce tumour cell secretion of HMGB1. Paclitaxel, but not carboplatin, was found to induce ICD through the release of HMGB1 and activation of TLR-4-dependent and -independent pathways in ovarian cancer (Lau et al. 2019). Single agent chemotherapy treatment did not significantly upregulate HMGB1 on OGJ cells, however, their combination as part of the FLOT and CROSS CT regimen did, further supporting a rationale for combining different chemotherapies with distinct mechanisms of action to not only reduce the development of chemoresistance via cancer cell intrinsic genetic mutations but also due to their ability to increase immunogenic cell death in combination.

Calreticulin is a pre-apoptotic marker which translocates from the endoplasmic reticulum to the cell surface as a result of endoplasmic reticulum stress (Martins et al. 2010). Membrane exposure of calreticulin acts as a phagocytic signal and attracts antigen presenting cells to the tumour site and promotes their subsequent activation and maturation (Martins et al. 2010). In our study, 5-FU, docetaxel and cisplatin increased calreticulin expression on the surface of viable OE33 cells, however, the combination chemotherapy regimens FLOT, CROSS CT or MAGIC did not significantly increase calreticulin. In contrast, oxaliplatin induced cell surface calreticulin expression in colorectal cancer (Wang et al. 2018) and murine lung carcinoma cell lines (Sun et al. 2019), while docetaxel induced calreticulin cell surface expression in breast, prostate and colorectal cancer cell lines in vitro*.* Collectively, our study and previous studies highlight that the ability of chemotherapies to induce ICD depends on the specific combination of drugs used which likely alters the type and level of genotoxic stress within the cell and ultimately dictates whether the cell will die in an immunogenic manner or an immunologically silent manner. In addition, the specific organ of origin and genetic landscape of the cancer cell lines likely plays an important role in whether a chemotherapy agent or regimen will induce ICD (Haruna et al. 2020).

It is well established that tumour cells and stromal cells within the OGJ TME secrete an array of immunosuppressive soluble factors (Lin et al. 2016). Chemotherapy and radiotherapy have a substantial effect on the TME contexture and may propagate, dampen, or skew an immunosuppressive milieu toward an immune competent TME (Davern and Lysaght 2020; Donlon et al. 2021). This study aimed to encapsulate the effect of these chemotherapy regimens on the OGJ cell line secretome, reflective of epithelial cells only, and the whole TME using tumour explants and their subsequent effects on T cell activation. Both the in vitro and ex vivo culture systems resulted in a significant reduction in the percentage of CD27^+^ T cells following treatment with FLOT, CROSS CT and MAGIC OGJ cell conditioned media as well as treatment with post-FLOT and post-CROSS CRT TCM. These findings were further supported through immunophenotyping of T cells in OGJ patient-derived peripheral blood and tumour tissue, which also demonstrated that the frequencies of CD27^+^ T cells were significantly lower post-FLOT and post-CROSS CRT in tumour tissue. The implications of these findings on anti-tumour immunity in OGJ patients are not clear as the role of CD27 signalling in cancer remains conflicting, CD27 signalling either leads to improved T cell function or to T cell dysfunction, likely dependent upon the amount, duration, and timing of the expression of the CD27 ligand (CD70) (Nolte et al. 2009; Matter et al. 2006), offering a likely explanation to the conflicting evidence for CD27–CD70 interaction in promoting or inhibiting anti-cancer immunity. CD27, is a co-stimulatory immune checkpoint receptor and a member of the tumour necrosis factor receptor superfamily and functions in eliciting T cell responses (Borst et al. 2005). CD27 signalling promotes cell T cell survival, enhances antigen receptor-mediated proliferative signals, and increases effector function (Borst et al. 2005). In addition, CD27 signalling increases the production of the T-cell growth/survival factor interleukin-2, which is also a key survival factor for regulatory T cells (Peperzak et al. 2010). Claus et al., demonstrated using fibrosarcoma, colon adenocarcinoma and melanoma tumour-bearing wild-type mice, that the CD27–CD70 interaction increased the frequency of regulatory T cells, reduced tumour-specific T-cell responses, increased angiogenesis, and promoted tumour growth (Claus et al. 2012). Moreover, this study reported that CD27 signalling reduced apoptosis of regulatory T cells in vivo and induced CD4^+^ effector T cells to produce interleukin-2, a key survival factor for regulatory T cells (Claus et al. 2012). Consequently, the frequency of regulatory T cells and growth of solid tumours were reduced in *Cd27*deficient mice and in wild-type mice treated with monoclonal antibody to block CD27 signalling (Claus et al. 2012; French et al. 2007). Constitutive expression of CD70 on tumour cells has been documented in cancer in contrast with its limited expression in normal healthy individuals (Wischhusen et al. 2002; Yang et al. 2007). Expression of CD70 in brain (Wischhusen et al. 2002), renal (Law et al. 2328) and lymphoma tumours (Wischhusen et al. 2002; Jak et al. 2009) has been shown to correlate with a more aggressive phenotype including increased metastasis (Sloan et al. 2004), regulatory T cell formation (Yang et al. 2007; Jak et al. 2009), or death of effector cells (Wischhusen et al. 2002). In contrast, injection of CD27-activating antibody improved tumour rejection (French et al. 2007; Roberts et al. 2010) and *cd70* transgenic mice were protected against poorly immunogenic tumour cells (Arens et al. 2004; Keller et al. 2008). Given the reported conflicting roles of CD27 in both promoting and/or hindering anti-tumour immunity in the literature, the downregulation of CD27 on T cells following neoadjuvant treatment in OGJ patients in this study may be detrimental or beneficial in promoting anti-tumour immunity and may likely be dependent on the tumour immune contexture within patients. Further studies are warranted to determine its biological significance and therapeutic potential in OGJ.

Interestingly, a significant increase in the percentage of CD69^+^ T cells was observed following treatment with FLOT and CROSS CT OGJ cell line conditioned media. These findings were also supported by the ex vivo culture experiments, where the percentages of CD69^+^ T cells were significantly increased following treatment with post-FLOT and post-CROSS CRT tumour tissue conditioned media. Moreover, the frequencies of CD69^+^ T cells were significantly higher in the circulating peripheral blood and infiltrating tumour tissue post-CROSS CRT but not post-FLOT in OGJ patients. Co-stimulatory CD69 is a type II glycoprotein with important functions in regulating inflammation through T cell migration, retention in tissues, and plays an important role sustaining T cell activation, proliferation, cytolytic activity (Borrego et al 1999) but also in inducing the exhaustion of tumour-infiltrating T cells perhaps as a result of prolonged T cell activation promoted by CD69 signalling in the T cells (Mita et al. 2018). Mita et al., demonstrated in *CD69*^*–/–*^ mice using 4T1-luc2 murine breast cancer models, a significant reduction in tumour growth and metastasis, increased levels of tumour-infiltrating lymphocytes and a significant reduction in T cell exhaustion and enhanced IFN-γ production compared with wild-type controls (Mita et al. 2018). Additionally, anti-CD69 monoclonal antibody treatment attenuated the T-cell exhaustion and tumour progression in tumour-bearing mice (Mita et al. 2018). Considering our findings in the context of the study by Mita et al. (2018) FLOT and CROSS regimens may enhance anti-tumour immunity through upregulation of CD69 however, this may also contribute to the induction of T cell exhaustion in OGJ in a chemotherapy-driven manner as according to the literature, CD69 signalling not only promotes sustained T cell activation and proliferation but also results in T cell exhaustion (Borrego et al. 1999; Mita et al. 2018). Further studies are certainly warranted to interrogate this theory and elucidate whether CD69 may be a viable novel target for cancer immunotherapy in OGJ in combination with FLOT or CROSS regimens to prevent tumour immune escape mediated by T cell exhaustion. Furthermore, this might suggest that addition of ICIs with chemotherapy may help prevent CD69-driven exhaustion.

Using ex vivo and in vitro models, the findings from this study demonstrated that the post-FLOT and post-CROSS chemotherapy tumour cell secretome and tumour biopsy secretome significantly increased the frequencies of effector memory and terminally differentiated effector memory CD8^+^ T cell phenotypes. Effector T cells elicit direct cytotoxicity against transformed cells via Fas/FasL and granzyme/perforin (Reiser and Banerjee 2016). Terminally differentiated effector memory T cells are highly cytotoxic, rapidly release effector molecules and quickly differentiate into effector T cells upon antigen rechallenge (Reiser and Banerjee 2016). In addition, complementary findings in this study demonstrated that FLOT and CROSS CT OGJ cell line conditioned media increased the cytotoxic potential of T cells in vitro. Collectively, these results may suggest that FLOT- and CROSS CT-induced alterations in the TME potentiate T cell activation.

Several soluble factors within the TME create “tolerizing” conditions and thereby help the tumour to evade antitumoral immune responses (Lapeyre-Prost et al. 2017). Therefore, our study investigated the effect of conditioned media post-chemo(radio)therapy on T cell activation status and interestingly found that the percentage of CD4^+^CD69^+^ T cells and effector CD8^+^ T cells negatively correlated with the levels of VEGFA present in the TCM. VEGFA is already known for its major role in promoting angiogenesis but plays a key factor in tumour-induced immunosuppression through its pleiotropic effects in driving the proliferation of immunosuppressive cell types, limiting T-cell recruitment into tumours, and promoting T-cell exhaustion (Lapeyre-Prost et al. 2017). The findings from this study suggest that VEGFA may play an important role in attenuating T cell activation in OGJ tumours and perhaps anti-VEGFA therapies may be an attractive therapeutic to combine with chemoimmunotherapy strategies. Ramucirumab, an anti-VEGFR2 monoclonal antibody which blocks the binding of VEGF-A, -C and -D) is approved in combination with paclitaxel based on findings from the phase 3 RAINBOW trial which demonstrated that combining ramucirumab with paclitaxel significantly prolonged survival compared with paclitaxel alone in previously treated or advanced OGJ and GC tumours (9.63 versus 7.26 months, respectively) (Vita et al. 2019).

Interestingly, the killing capacity of lymphocytes from treatment-naïve OGJ patients was enhanced in the presence of post-FLOT and post-CROSS tumour cell secretome, suggesting that the tumour cell secretome following FLOT or CROSS CT treatment may not only be potentiating T cell activation status and cytotoxic potential but also increasing the functional capacity of lymphocytes to kill OGJ cells. This may be due to the presence of DAMPs in the post-FLOT or post-CROSS tumour cell secretome, which may be stimulating T cell activity or perhaps analytes present in the tumour cell secretome may be altering the cancer cells making them more vulnerable to lymphocyte-mediated killing.

We acknowledge that the number of patients within our study is low given that OGJ is a rare cancer type in Ireland, which has a new diagnosis rate of under 450 cases per year. In addition, 50% of newly diagnosed OGJ patients present with metastatic disease and are treated with palliative care instead of receiving neoadjuvant therapy making these patients ineligible for this study. Therefore, this is a limiting factor that restrains us from drawing firm conclusions from this study. However, we do believe these findings offer an interesting preamble for future studies and highlight potential immunomodulatory effects of FLOT and CROSS chemotherapy regimens which could be leveraged via combining with immunotherapy to boost response rates in OGJ.

Our in vitro and ex vivo data strongly suggest that FLOT and CROSS chemotherapy regimens enhance the immunogenicity of OGJ tumours via increased immunostimulatory marker expression and subsequently enhanced OGJ lymphocyte-mediated killing of OGJ cells. Additionally, our findings highlight that FLOT and CROSS regimens upregulate CD69 expression on T cells, which despite being an early marker of T cell activation also plays an important role in driving T cell exhaustion and may represent an attractive therapeutic target that could be combined with FLOT and CROSS regimens to enhance anti-tumour immunity, prevent immune exhaustion and ultimately promote tumour eradication in OGJ patients.

## Supplementary Information

Below is the link to the electronic supplementary material.

Supplementary file1 (DOCX 3058 KB)
