# Supplementary material for: FLOT and CROSS chemotherapy regimens alter the frequency of CD27+ and CD69+ T cells in oesophagogastric adenocarcinomas: implications for combination with immunotherapy
Source: J Cancer Res Clin Oncol. 2022 Aug 20;149(7):3753–74. doi: 10.1007/s00432-022-04283-9 (PMC10314858; doi:10.1007/s00432-022-04283-9)
Supplement: Supplementary file 1 — Supplementary file1 (DOCX 3058 KB) [file 432_2022_4283_MOESM1_ESM.docx]

**Supplemental**

**
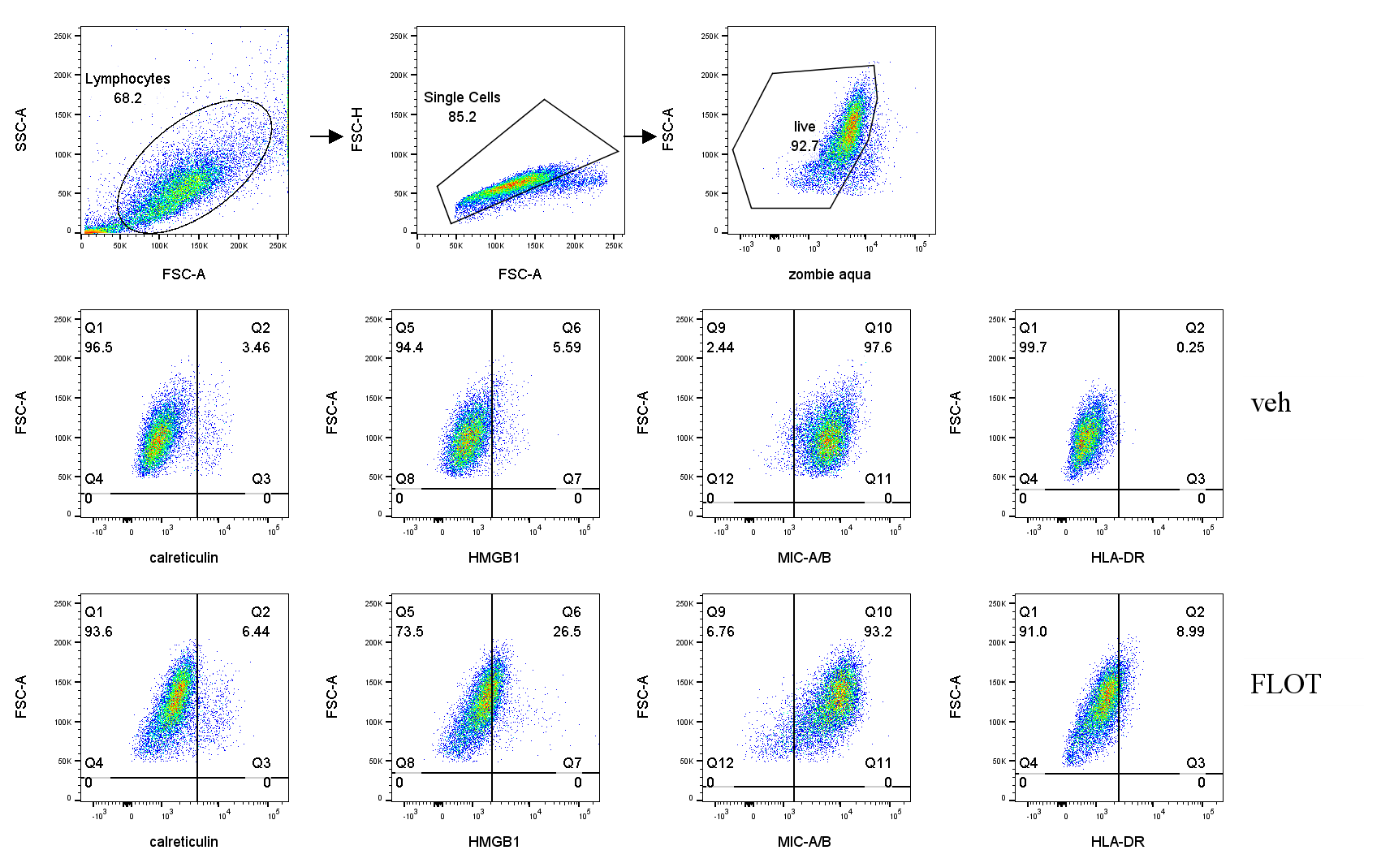
**

**Fig. S1. Gating strategy for assessing expression of DAMPs on the surface of OGJ cells by flow cytometry.** Gate 1 included all cells in the FSC versus SSCA plot, doublet cells were then excluded using FSC-H versus FSC-A plot, dead cells were excluded using zombie aqua viability marker. The surface expression of calreticulin, HMGB1, MIC-A/B and HLA-DR on OGJ cells was then assessed on this live, doublet excluded population. Representative dot plots are shown for veh and FLOT treated OE33 cells *in vitro*.

**
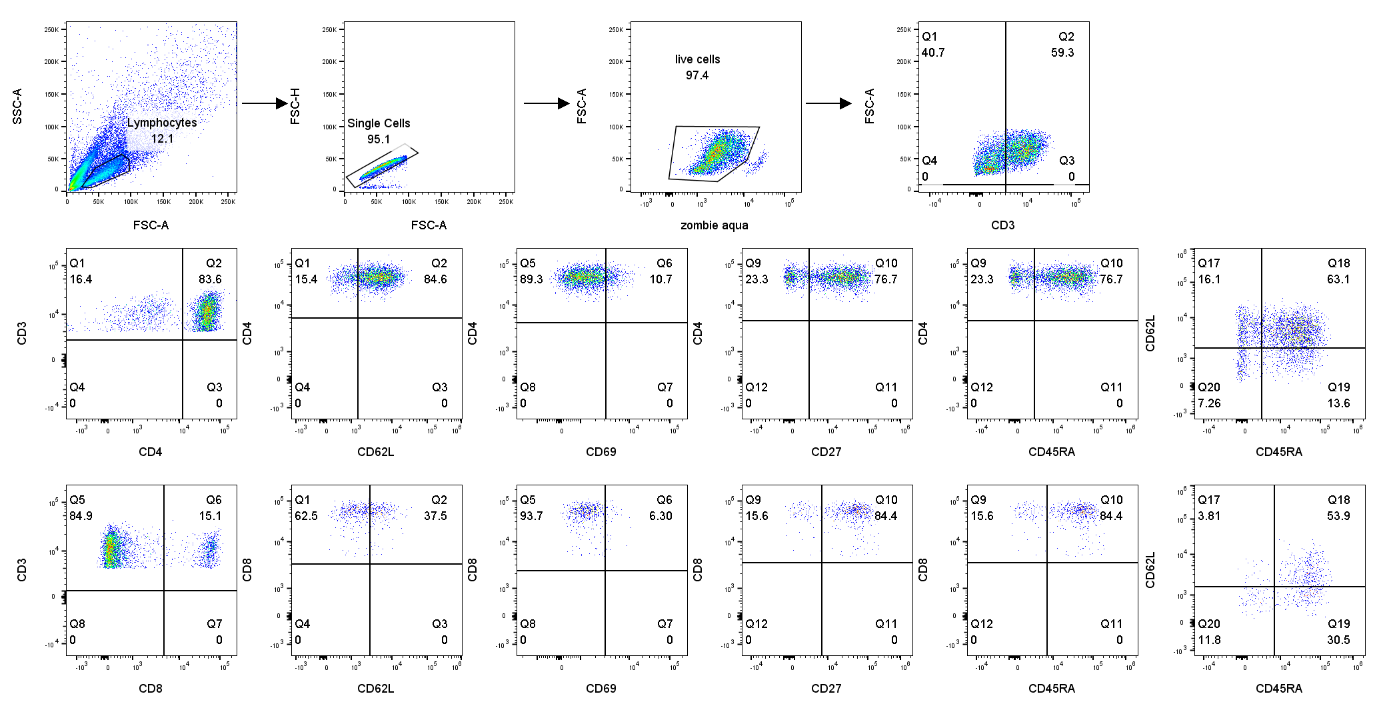
**

B

A


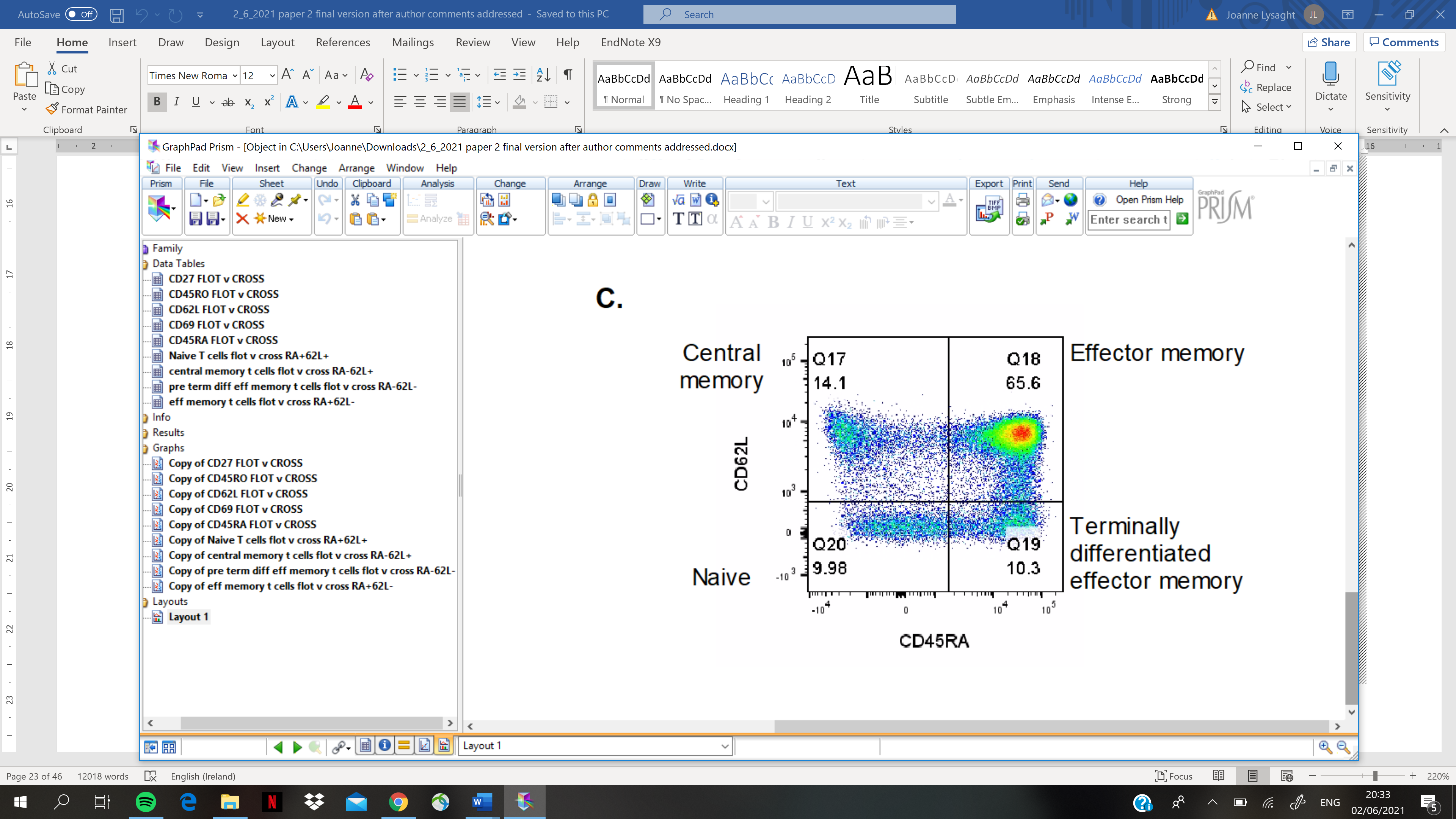


**Fig. S2. Gating strategy for assessing expression of activation markers on T cells surfaces and T cell differentiation states by flow cytometry.** Gate 1 included all cells in the FSC versus SSCA plot, doublet cells were then excluded using FSC-H versus FSC-A plot, dead cells were excluded using zombie aqua viability marker. The surface expression of CD62L, CD69, CD27 and CD45RA was assessed on CD3^+^CD4^+^ cells and CD3^+^CD8^+^ cells. The percentage of CD3^+^CD4^+^ cells and CD3^+^CD8^+^ cells co-expressing CD45RA^+^CD62L^+^ (naïve T cells), CD45RA^-^CD62L^+^, (central memory T cells), CD45RA^-^CD62L^-^ (effector memory T cells) and CD45RA^+^CD62L^-^ (terminally differentiated effector memory T cells) was also assessed by flow cytometry (A). Representative dot plots are shown for each marker. Representative dot plot showing the characterisation of naïve, central memory, effector memory and terminally differentiated T cells following treatment with TCM (B).

**
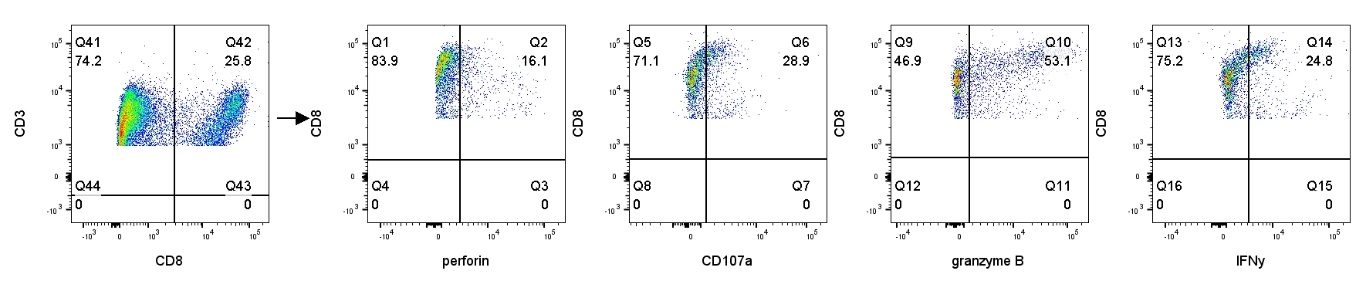

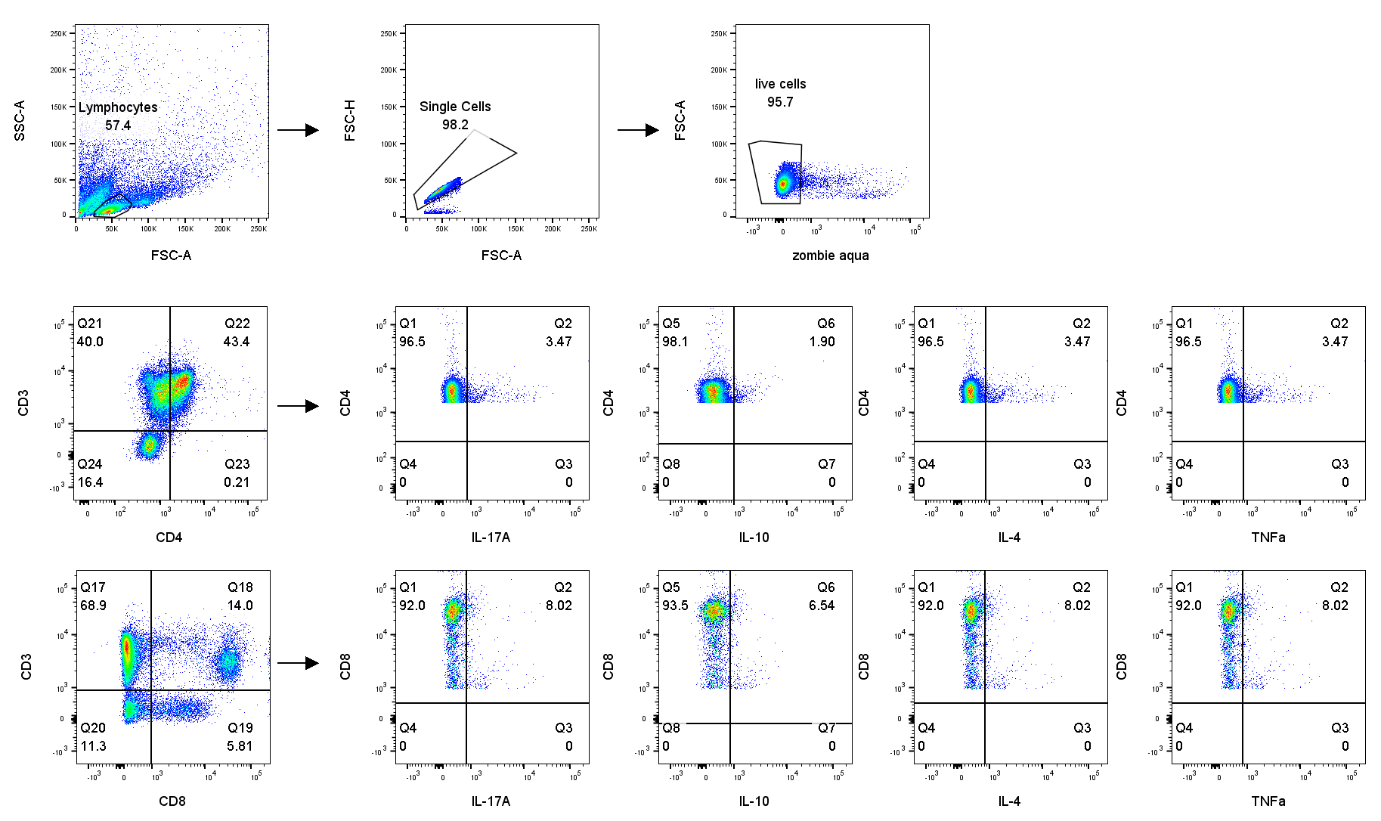
**

**Fig. S3. Gating strategy for assessing cytokine production by T cells by flow cytometry.** Gate 1 included all cells in the FSC versus SSCA plot, doublet cells were then excluded using FSC-H versus FSC-A plot, dead cells were excluded using zombie aqua viability marker. The intracellular surface expression of IL-17A, IL-10, IL-4 and TNF-α was assessed in CD3^+^CD4^+^ cells and CD3^+^CD8^+^ cells. Intracellular perforin expression, extracellular CD107a expression and intracellular granzyme B and IFN-γ was assessed on CD3^+^CD8^+^ cells by flow cytometry. Representative dot plots are shown for each marker.
